# The Participation of the Intrinsically Disordered Regions of the bHLH-PAS Transcription Factors in Disease Development

**DOI:** 10.3390/ijms22062868

**Published:** 2021-03-11

**Authors:** Marta Kolonko-Adamska, Vladimir N. Uversky, Beata Greb-Markiewicz

**Affiliations:** 1 Department of Biochemistry, Molecular Biology and Biotechnology, Faculty of Chemistry, Wroclaw University of Science and Technology, Wybrzeze Wyspianskiego 27, 50-370 Wroclaw, Poland; marta.kolonko@pwr.edu.pl; 2 Department of Molecular Medicine, USF Health Byrd Alzheimer’s Research Institute, Morsani College of Medicine, University of South Florida, Tampa, FL 33612, USA; vuversky@usf.edu; 3 Laboratory of New Methods in Biology, Institute for Biological Instrumentation, Russian Academy of Sciences, Federal Research Center “Pushchino Scientific Center for Biological Research of the Russian Academy of Sciences”, 142290 Pushchino, Moscow Region, Russia

**Keywords:** disease-associated mutation, intrinsically disordered region (IDR), liquid-liquid phase separation (LLPS), post-translational modifications (PTM), AhRR, AhR, Single-Minded Protein 1 (SIM1), SIM2, Hif-2α, NPAS4, ARNT2, BMAL1, D^2^P^2^, disorder prediction, LLPS prediction, cancer, HuVarBase, Catalogue of Somatic Mutations in Cancer (COSMIC), catGranule, PScore, STRING, Waltz

## Abstract

The basic helix–loop–helix/Per-ARNT-SIM (bHLH-PAS) proteins are a family of transcription factors regulating expression of a wide range of genes involved in different functions, ranging from differentiation and development control by oxygen and toxins sensing to circadian clock setting. In addition to the well-preserved DNA-binding bHLH and PAS domains, bHLH-PAS proteins contain long intrinsically disordered C-terminal regions, responsible for regulation of their activity. Our aim was to analyze the potential connection between disordered regions of the bHLH-PAS transcription factors, post-transcriptional modifications and liquid-liquid phase separation, in the context of disease-associated missense mutations. Highly flexible disordered regions, enriched in short motives which are more ordered, are responsible for a wide spectrum of interactions with transcriptional co-regulators. Based on our in silico analysis and taking into account the fact that the functions of transcription factors can be modulated by posttranslational modifications and spontaneous phase separation, we assume that the locations of missense mutations inducing disease states are clearly related to sequences directly undergoing these processes or to sequences responsible for their regulation.

## 1. Introduction

### 1.1. bHLH-PAS Proteins

The basic helix–loop–helix/Per-ARNT-SIM (bHLH–PAS) proteins are an important class of transcription factors (TFs) responsible for the regulation of developmental and physiological events occurring in mammals [1]. Representatives of this family perform a wide spectrum of functions, starting with the Aryl hydrocarbon receptor (AHR) acting as receptor for environmental stimuli including highly toxic dioxins [2] to Clock and Aryl hydrocarbon receptor nuclear translocator-like protein 1 (ARNTL1, Bmal1) regulating circadian rhythms of the organism [3], and to Hypoxia inducible factor 1α (Hif-1α) [4], acting as a specific oxygen sensor in cells. In hypoxia conditions, Hif-1α trans-locates from cytoplasm to the nucleus, binds to the Aryl Hydrocarbon Receptor Nuclear Translocator (ARNT), and induces the expression of genes related to angiogenesis, cell proliferation, glucose, and iron metabolism [5]. The incorrect control of these processes is commonly connected with the genesis of many diseases, including cancer, strokes, and heart diseases [4].

bHLH-PAS proteins are commonly divided into two classes based on their dimerization pattern, with proteins assigned to class I unable to form homodimers. Additionally, their expression is specifically regulated by physiological states and/or environmental signals. This class comprises mammalian AhR, Aryl hydrocarbon Receptor Repressor (AhRR), Single-Minded Protein 1 (SIM1), SIM2, Hif-1α, Hif-2α, Hif-3α, and Neuronal PAS-Domain Containing Protein 1 (NPAS1), NPAS2, NPAS4 and NPAS4 TFs. In contrast, the class II family members can homodimerize and serve as general partners for class I TFs. This class of proteins is expressed constitutively and comprises ARNT, ARNT2, BMAL1 and BMAL2 TFs. Importantly, only heterodimers formed by class I and class II proteins act as the functional TF complex and regulate gene expression [6,7].

**Figure 1:**
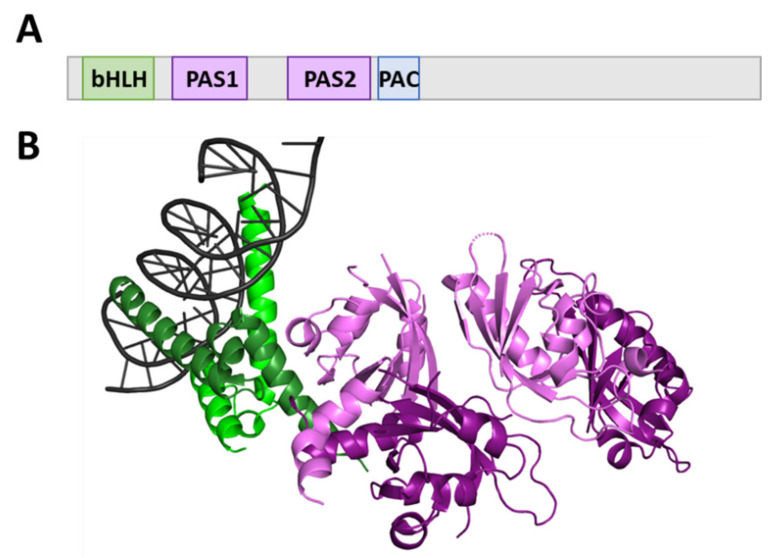
Structure organization of basic helix–loop–helix/Per-ARNT-SIM (bHLH-PAS) proteins. (**A**) The domain structure of bHLH-PAS proteins [12]; green indicates the bHLH domain, purple indicates PAS domains, and blue indicates PAS-associated C-terminal (PAC), respectively, (**B**) crystal structure of the heterodimeric NPAS3-ARNT complex with Hypoxia Response Element (HRE) DNA (PDB: 5SY7) [13]. The bHLH domain, responsible for DNA binding, is colored in green, whereas PAS-Domain Containing Protein 1 (PAS1) and PAS2 domains are colored in purple.

Despite mediating highly diversified signaling pathways, the domain organization of bHLH-PAS proteins is rather conserved. The bHLH domain, typically located at the N-terminus of the protein, is responsible for DNA binding and dimerization [8] (Figure 1A). It consists of two α-helices connected by a loop (Figure 1B) [9] and is followed by a PAS domain that comprises two structurally conserved regions: PAS1 and PAS2, separated by a poorly conserved link (Figure 1A) [1,10]. The PAS core is characterized by an antiparallel β-sheet surrounded by several α-helices (Figure 1B) [11]. While the PAS1 region is responsible for the selection of a dimerization partner and specificity of target genes activation [12], the PAS2 region binds to ligands/cofactors and is often connected to a single PAS-associated C-terminal (PAC) motif [10]. PAC is proposed to contribute to the PAS domain appropriate folding. Each binding event may affect protein conformation and, thus, its activity [12]. In contrast to defined domains located within the N-terminal part of bHLH–PAS proteins, their C-termini are characterized by a significant variability in primary structure and are considered as highly important and unique parts of the proteins responsible for the specific modulation of the bHLH–PAS protein action [12]. They usually comprise specific regions responsible for protein–protein interaction (PPI) known as transcription activation/repression domains (TADs/RPDs) [13,14]. Importantly, C-termini of most of the bHLH-PAS proteins were predicted as intrinsically disordered regions (IDRs) [15].

Being biologically active, IDRs and intrinsically disordered proteins (IDPs) do not possess unique stable tertiary structures in physiological conditions [16], thereby contradict the fundamental paradigm of biochemistry and structural biology stating that the unique function of a protein results directly from its unique tertiary structure [17]. Currently, more than 20–30% of eukaryotic proteins have been found to present features of IDPs, and over 70% of proteins involved in signal transduction cascades have long IDRs. IDPs were identified as important elements in a wide range of biological processes, such as cell cycle, cell differentiation, regulation of transcription, mRNA processing, and apoptosis control [18,19,20].

The lack of a defined structure is critical for IDP and IDR functionalities [19]. Interestingly, IDRs found in bHLH TFs were proposed to contribute directly to the evolution of complex multicellularity [21]. The conformational plasticity allows IDPs/IDRs to interact with several unrelated proteins/ligands, with such binding promiscuity seeming to be highly useful for the molecular recognition processes [22]. For this reason IDPs are commonly involved in one-to-many and in many-to-one interactions and can function as hub proteins responsible for the cross-talk of different pathways [23]. Often, IDRs contain Molecular Recognition Features (MoRFs), which are interaction-prone segments of protein disorder exhibiting molecular recognition and binding functions and facilitating interactions with physiological partners. MoRFs undergo a disorder-to-order transition as a result of interaction with specific partners and such binding-induced folding allows them to perform various biological functions [24]. Their extended conformation and low compactness make IDPs excellent targets for post-translational modifications (PTMs) and proteolytic degradation, which are typical means activity regulation in proteins [25].

IDPs/IDRs were shown to play an important role in the formation of self-assembled, membrane-less organelles (MLOs) through liquid–liquid phase separation (LLPS). Interestingly, although in some cases PPI could lead to LLPS formation, there are also instances where LLPS may prevent protein interactions [26,27,28]. In the context of TFs, it is very interesting to consider the putative role of LLPS in fast cellular responses to external stimuli [29]. The ability of protein to undergo the LLPS process may be regulated by a wide spectrum of PTMs and alternative splicing [30]. Recently, we discussed the disordered character of bHLH TFs and their propensities to LLPS [31]. Experimental data have provided evidence that MyoD belonging to bHLH TFs family, and disordered regions of TFs, such as Oct4 and Brd4, can form liquid condensates [32]. Regulation of the circadian clock by BMAL1 also partially occurs in discrete nuclear foci resembling phase separated droplets [33]. Proteome-wide analyses of disease-related mutations have shown that gain or loss of post-translational modification sites might contribute to various human diseases. Importantly, most PTMs are found in IDRs. In addition, more than 80% of proteins considered as responsible for oncogenesis in humans are enriched in IDRs [34]. The ability of IDR-containing proteins to form multivalent, weak, and transient interactions underlie the ability of particular proteins to undergo LLPS. IDRs are often depleted in hydrophobic residues; however these residues can represent adhesive elements in phase-separating IDRs and mediate condensation upon changes in temperature [26]. In turn, repetitively distributed, highly, but oppositely, charged regions, short motifs such as YG/S-, FG-, RG-, GY-, KSPEA-, SY-, and Q/N-rich regions might be engaged in the formation of the multivalent interactions between condensate components [35]. Highly charged and flexible IDRs are in fact frequently identified as scaffold proteins and undergo spontaneous LLPS. Furthermore, they are essential for the structural integrity of a condensate [36].

As IDRs are suggested as the most important regulatory regions for proteins, we were interested in finding out if there is a pattern of the distribution of disease associated missense mutations among ordered and disordered regions in bHLH-PAS protein family members. Are the missense mutations observed more frequently in IDRs prone to PTMs, LLPS or aggregation?

To address this problem, we decided to analyze the known aa missense mutations listed in the HuVarBase database and to compare their localizations with the localizations of documented PTMs (PhosphoSitePlus database) and predicted MoRFs (Anchor server), simultaneously with the in silico analysis of protein’s LLPS (catGranule and PScore servers) and amylogenic propensity (Waltz predictor). Based on the results, we assume that most of the disease-associated missense mutations are localized in IDRs of analyzed and selected bHLH-PAS family representatives.

The aim of this work was to produce a foundation for future experimental studies dedicated to the analysis of the effects of mutations affecting bHLH-PAS TFs’ functionality.

### 1.2. bHLH-PAS Proteins and Diseases

#### 1.2.1. AhR and AhRR

AhR, best known as a mediator of environmental pollutant toxicity, also contributes to the proper functioning of the liver, cardiovascular, immune, and reproductive systems [37]. AhR is also related to normal skin formation during fetal development and to pathological states such as epidermal wound healing and skin carcinogenesis [38]. Recently, AhR has been recognized as an important modulator of diseases driven by immune/inflammatory processes [39]. The ligand-bound AhR trans-locates to the nucleus, where it mediates the biological response to toxins resulting in wasting syndrome, hepatotoxicity, teratogenesis, and tumor promotion [2]. Activation of AhR was linked to chronic kidney and cardiovascular diseases [37]. The overexpression and constitutive AhR activation have been assigned to various types of tumors [40] including brain tumors, such as gliomas, meningiomas, medulloblastomas, and neuroblastomas [41]. Furthermore, AhR activation is linked to renal damage, diabetic nephropathy, and urinary system-associated cancers [37]. AhR can heterodimerize with ARNT to function as a co-regulator of the estrogen signaling pathway mediated by the estrogen receptor (ER) [42] and is considered as responsible for the connection between inflammation process and breast cancer [43].

Interestingly, AhR self-regulates its activity by activation of the repressor, AhRR. In comparison to AhR, present in most tissues, AhRR is characterized by high tissue specificity. The highest concentration of this protein was observed in the testis, lung, spleen, heart, and kidney [44]. The repressor competes with AhR for binding to the ARNT and forms an inactive AhRR/ARNT heterodimer [43]. AhRR is not able to bind to AhR ligands because it does not possess the PAS2 domain in the N-terminal region. Additionally, AhRR contains the C-terminal trans-repression domain (instead of the transactivation domains in the AhR C-terminus), that allows binding of the corepressors involved in a negative regulatory loop [45]. Zudaire et al. [46] demonstrated downregulation of AhRR expression in human malignant tissues of different anatomical origin, such as colon, breast, lung, stomach, cervix, and ovary. Genetic polymorphisms of AhRR were also related to enhanced susceptibility to advanced endometriosis [47,48]. Interestingly, it was observed that AhRR splice variant is able to inhibit transcription activated by Hif-1, which is essential for cancer progression [49].

#### 1.2.2. Single Minded Protein (SIM)

The mammalian SIM exists as two homologs that are encoded by two different genes: SIM1 and SIM2, with a high level of amino acid identity shared by their N-terminal parts (90% identity in the bHLH and PAS domains), and a high level of diversity in their C-terminal parts [50]. While SIM1 is responsible for the activation of specific genes’ expression, SIM2 is defined as a transcription inhibitor. The opposite transcriptional effect results from the presence of two repression domains within the SIM2 C-terminal sequence [51,52]. This example confirms the importance of the C-terminal region for the functions and activities of bHLH–PAS proteins [12]. SIM1 dimerizes with ARNT and activates transcription of specific genes related to the development, terminal differentiation, and post-development functioning of neuronal cells, especially in the paraventricular nucleus of the hypothalamus (PVN) [53]. Importantly, PVN is responsible for several autonomic processes, including response to stress, metabolism control, growth, reproduction and appetite regulation [53]. Since the SIM1 plays a role in the long-term regulation of food intake and energy expenditure [54], its reduced activity is manifested phenotypically as profound obesity and increased linear growth. The weight gain is connected to high food consumption, since measured energy expenditure is usual [54]. It was shown that SIM1 haploinsufficiency in mice induces hyperphagia (abnormally increased appetite for consumption of food) [55] leading to obesity and developmental abnormalities of the brain [56]. It has been shown that transgenic mice with overexpressed SIM1 are resistant to diet-induced obesity, which supports a post developmental, physiologic role for SIM1 in feeding regulation [57]. Induced SIM1 overexpression contributes to decreased food intake [58].

#### 1.2.3. Hypoxia Inducible Factor 2α (Hif-2α)

Functional hypoxia inducible factors are heterodimers comprising one of the three known α subunits regulated by oxygen (Hif-1α, Hif-2α and Hif-3α), and constitutively expressed ARNT (known also as Hif-1β) [59]. For the first time, Hif-2α, also known as endothelial PAS-1 protein (EPAS1), was isolated from the endothelial cells [60]. Hif-2α shares approximately 50% sequence identity with the ubiquitously expressed Hif-1α, and the activities of both proteins are regulated by oxygen level. Under normoxic conditions, two proline residues of the oxygen-dependent degradation domain located in the C-termini of Hif-1α/Hif-2α are hydroxylated and targeted to the ubiquitin–proteasome (26S) pathway for degradation. Additionally, hydroxylation of the arginine residues prevents protein interaction with coactivator protein p300 [61]. Similar to Hif-1α, Hif-2α was shown to induce the expression of genes stimulating cell cycle progression, proliferation, apoptosis promotion, autophagy and angiogenesis [59]. Furthermore, Hif-2α regulates erythropoietin level and is involved in embryonic development and metastasis [62,63]. Interestingly, Hif-2α is localized within the nucleus in the form of puncta, whereas Hif-1α is distributed homogeneously in the nucleus. Distinct subnuclear localizations of both proteins were proposed to contribute to the different regulations and activities of these two TFs [64]. Importantly, Hif-2α shuttling is regulated by phosphorylation [65]. Some studies of kidney cancer suggested an oncogenic role for Hif-2α, which is in contrast to Hif-1α that manifested tumor suppressor properties [66]. Missense mutations within the bHLH and PAS domains of Hif-1α/Hif-2α proteins have been linked to pathogenesis of various cancers, such as stomach adenocarcinomas, endometrial carcinomas, brain gliomas, lung adenocarcinomas, hepatocellular carcinomas and skin melanomas [61]. The Gly537 residue located close to the primary oxygenation site is conserved among all known Hif-2α proteins, whereas mutation of this residue results in the familial erythrocytosis characterized by an increased number of red blood cells. The familial erythrocytosis symptoms are headaches, dizziness, nosebleeds, and shortness of breath. Additionally, an excess of red blood cells increases the risk of developing abnormal blood clots [67].

#### 1.2.4. Neuronal PAS-Domain Containing Protein 4 (NPAS4)

Initially, it was shown that the NPAS4 protein is expressed and acts mainly in the nervous system [68]. However, later studies have shown that NPAS4 is also expressed in β cells of pancreatic islets, which significantly affects pancreatic cells. In this case, NPAS4 expression is induced by endoplasmic reticulum stressors and prevents the death of β-cells [69,70]. In the nervous system, NPAS4 is responsible for the regulation of the development of GABAergic inhibitory neurons [71]. NPAS4 was shown to be able to inhibit seizure attacks in pilocarpine-induced epileptic rats [72]. Importantly, increased levels of NPAS4 expression have been linked to brain protection in focal and generalized ischemic strokes of the brain, where it prevented necrosis and led to cell apoptosis [73,74]. It was also shown that NPAS4 is involved in the structural plasticity of the nervous system and plays an important role in the formation of long-term memory. Its expression is highly induced during the learning process [75,76]. Interestingly, NPAS4 overexpression can reverse tau protein aggregation [77]. Finally, NPAS4 expression was also detected in endothelial cells, where, similar to pancreatic β-cells, it promoted pro-angiogenic cell functions, such as migration or sprout formation [78].

For human NPAS4, a second isoform of NPAS4 comprising residues 1–234 (only bHLH and PAS-1 domains) with V234G substitution was proposed. However, there is no evidence for this isoform at the protein translation level, and its function is not known [79]. To date, only few dimerization partners for NPAS4 have been identified, such as ARNT and ARNT2, which are the general partners for the class I bHLH-PAS TFs in the brain [80] and the melanoma-associated antigen D1 (MAGED1), which is expressed ubiquitously in both developing and adult tissues, but is particularly abundant in the brain. MAGED1 participates in various signaling pathways, including apoptosis and differentiation of the neuronal precursors, periodicity stabilization in the circadian rhythm, and learning and memory formation [81]. As shown, NPAS4 developmental downregulation in the prefrontal cortex caused behavioral abnormalities observed in neurodevelopmental disorders, such as schizophrenia and autism [82]. NPAS4 was also linked to a number of other serious psychiatric disorders, including depression, Huntington’s disease, Down syndrome, and various neurodegenerative diseases (e.g., Alzheimer’s disease) [77].

#### 1.2.5. Aryl Hydrocarbon Receptor Nuclear Translocator 2 (ARNT2) and BMAL1

ARNT2 is a representative of the class II bHLH-PAS TFs. It is constitutively expressed and acts as general heterodimerization partner for multiple class I bHLH-PAS members, including SIM1 [83] and NPAS4 [84,85]. In contrast to the ARNT, which is expressed equally in all tissues and interacts with a wide spectrum of physiological partners (ARNT is indispensable for AHR and Hif signaling) [86], ARNT2 is expressed mainly in the brain (in the developing central nervous system (CNS)), kidney, urinary tract, and embryos [87,88]. ARNT2 deficiency leads to secondary microcephaly within the first few months of human life with a specific frontal and temporal lobe hypoplasia [89]. Secondary microcephaly indicates a progressive neurodegenerative condition caused by a decreased number of dendritic connections and/or reduced neuron activity [90]. The hypothalamic insufficiency can cause obesity, diabetes, and is often combined with pituitary hormone deficiency [89]. The latter seems to be consistent with a key role of ARNT2 in the development of specific neurosecretory neurons in the human hypothalamus [89]. Some ARNT2 mutants are also considered as causing hyperphagic obesity, diabetes, and hepatic steatosis [91]. ARNT2 was also shown to act as an important component of a protein complex located at a node of the TF network controlling glioblastoma cell aggressiveness [92].

BMAL1, together with its heterodimerization partner CLOCK, creates the core of the regulatory mechanism of mammalian circadian rhythms. The C-terminally located TAD of BMAL1 acts as a regulatory hub interacting with positive/negative transcriptional regulators in a circadian time-dependent manner to control the activation state of CLOCK-BMAL1 dimer [93]. The conformational switch of TAD is caused by cis/trans isomerization around a highly conserved W624-P625 imide bond [94]. BMAL1 polySUMOylation leads to its ubiquitination and binding of CREB-binding protein (CBP) that potentiates its transcriptional activity. Formation of nuclear bodies containing BMAL1/CBP provides transcriptionally active sites for target genes [33] and supports our thesis about the putative role of BMAL1 in LLPS formation. Similar to other bHLH-PAS TFs, BMAL1 is a shuttling protein [95]. Its localization signal activities are regulated by PTMs, e.g., phosphorylation [96]. BMAL1 was also shown to stimulate the translation process by interacting with the translational machinery in the cytosol, which was possible only after S42 phosphorylation [97]. Geyfman et al. [98] reported that the circadian variations in DNA sensitivity to UVB-induced damage depended on BMAL1 activity that directly connects circadian mechanisms with the epidermal carcinogenesis.

## 2. Results

To date, the structural characterization of bHLH-PAS TFs was limited to their bHLH-PAS regions, whereas no structural information is available for their C-terminal regions. This lack of structural knowledge can be explained by the difficulties associated with the expression and purification of the full-length proteins, caused by the presence of long disordered C-termini. We have discussed this research area in detail previously [15]. Curiously, all previously published data on the analysis of the missense mutations linked to cancers were limited to the bHLH-PAS domains of the selected bHLH-PAS members (Hif-1α and Hif-2α) [61].

Taking into account the connection of bHLH-PAS TFs with some serious disorders discussed in the previous sections, we asked a question about the localizations of known missense mutations associated with various diseases within the entire proteins, including their IDRs.

### 2.1. AhR and AhRR

**Figure 2:**
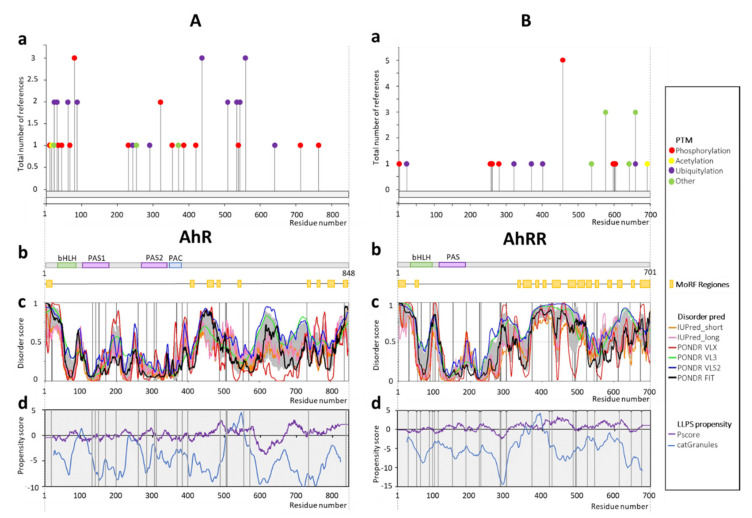
Schematic presentation of results for (**A**) AhR (P35869) and (**B**) AhRR (A9YTQ3) analysis. (**a**) Post-translational modifications based on PhosphoSitePlus server [99]; (**b**) the domain structure of protein: green indicates the bHLH domain (27–80aa AhR; 28–81aa AhRR), purple represents PAS domains (111–181aa PAS1, 275–342aa PAS2 AhR; 112–182aa PAS AhRR), whereas blue indicates PAC (348–386aa PAC AhR). Predicted MoRFs [100] are indicated as orange rectangles, (**c**) D^2^P^2^ database disorder regions predictions based on the protein amino acids sequence (find the legend in the plot for description). Grey shadow presents the averaged disorder profile, and a score over 0.5 indicates a high probability of disorder. Positions of disease-linked mutations are marked as black vertical lines (listed in HuVarBase database [101], Supplementary Materials), (**d**) liquid–liquid phase separation (LLPS) propensity predictions based on catGranules (blue line) [102] and PScore (purple line) [103] servers; positions of disease-linked mutations are marked as black vertical lines (listed in HuVarBase database [101], Supplementary Materials).

According to the PhosphoSitePlus, most of the documented PTMs (Figure 2(Aa)) are located within the disordered regions of AhR, which are predicted at the short N-terminal fragment preceding the bHLH domain (residues 1–26), the linker between PAS1 and PAS2 domains (residues 182–274) and a long C-terminal region of the protein (residues 387–848) (Figure 2(Ab,c)). In these regions, the presence of MoRFs was also predicted (Figure 2(Ab)). In contrast, all the regions corresponding to the conserved domains were predicted as highly ordered (Figure 2(Ab,c)), which is typical for bHLH-PAS proteins. The missense mutations in IDRs are linked mainly to large intestine cancer (T199P, P260L, N505S, T507I, P838S), soft tissue cancer (R554K), thyroid cancer (V570I), kidney cancer (E488K), and liver cancer (P18L) (Supplementary Materials). Importantly, results of the NetPhos 3.1 server prediction suggest many more phosphorylation sites (the most common PTM) in AhR than documented, for example, in the 100–200 aa region (Supplementary Materials).

The proximities of missense mutations (see Figure 2(Ac)) to the locations of known PTM sites (see Figure 2(Aa)) in some cases seem to be crucial for disease development. Prediction of the LLPS propensity resulted in a positive maximal score in the C-terminal fragment (residues 500–600) in the region enriched in the disease associated mutations (Figure 2(Ad)). The additional local positive maximum is observed in the linker between bHLH and the PAS domain, which is also predicted as locally disordered.

In the case of AhRR, all documented PTM sites (Figure 2(Ba)) and all MoRFs (Figure 2(Bb)) are located in IDRs (Figure 2(Bc)). Importantly, AhRR undergoes many rather uncommon PTMs, such as SUMOylation (see Figure 2(Ba), green points). AhRR, as transcription repressor, does not possess ligand binding PAS2 domain and is predicted as highly disordered not only at the N- and C-termini (residues 1–27 and 183–700), but also in the linker between the bHLH and PAS domains (residues 82–111) (Figure 2(Bc)). AhRR possesses a defined ordered structure only in the middle of the bHLH domain and in the entire PAS domain. LLPS propensity analysis shows a positive maximum for the central part of the protein (approximately residues 340–440) (Figure 2(Ad)) surrounded by various PTM sites. Furthermore, another maximum coincides with the segment of the disordered C-terminus. We can observe that AhRR C-terminus and the linker between its bHLH and PAS domains are enriched in the disease-associated mutations in reference to the ordered bHLH and PAS domains. Diseases associated with the mutations are represented mainly by intestine cancer (I226V, R230C, R285W, A300T, T419I, R485W, R491W, G494S, V553M, and D645H), skin cancer (P283S, A301V, and G427E), prostate cancer (R491Q and D645H) and liver cancer (C545F and A674S). The other single mutations are connected to endometrium cancer (A371T), CNS cancer (P189A), and esophagus cancer (G612S) (see Supplementary Materials). In the case of AhRR also, the NetPhos 3.1 server predicted more phosphorylation sites than documented (Supplementary Materials).

### 2.2. SIM1 and SIM2

**Figure 3:**
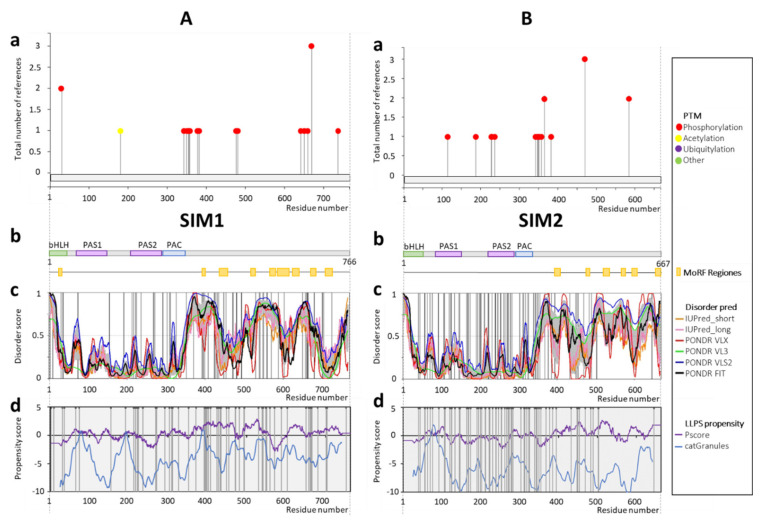
Schematic presentation of results for (**A**) SIM1 (P81133) and (**B**) SIM2 (Q14190) analysis. (**a**) Post-translational modifications based on PhosphoSitePlus server [99], (**b**) the domain structure of protein, green indicates the bHLH domain (1–63aa SIM1; 1–53aa SIM2), purple represents PAS domains (77–147aa PAS1 SIM1, 77–149aa PAS1 SIM2, 218–288aa PAS2 SIM1/2), whereas blue indicates PAC (292–335aa PAC SIM1/2). Predicted MoRFs [100] are indicated as orange rectangles, (**c**) D^2^P^2^ database disorder regions predictions based on the protein amino acids sequence (find the legend in the plot for description). Grey shadow presents the averaged disorder profile, and a score over 0.5 indicates a high probability of disorder. Positions of disease-linked mutations are marked as black vertical lines (listed in HuVarBase database [101], Supplementary Materials), (**d**) LLPS propensity predictions based on catGranules (blue line) [102] and PScore (purple line) [103] servers; positions of disease-linked mutations are marked as black vertical lines (listed in HuVarBase database [101], Supplementary Materials).

According to the disorder predictions, most of the documented PTMs (Figure 3(Aa)) and all predicted MoRFs (Figure 3(Ab)) of SIM1 are located at the long C-terminus (residues 336–766) (Figure 3(Ac)). An additional disordered region is predicted in the linker between the bHLH and PAS1 domains (residues 64–76) (Figure 3(Ac)). Prediction of phosphorylation sites by NetPhos resulted in positive scores for many sites along the whole protein (Supplementary Materials). bHLH and PAS domains, as well as several short regions observed in the C-terminus of SIM1 (residues 450–500 and 700–740, Figure 3(Ac)) are predicted as more ordered. Importantly, the short ordered regions in the middle of disordered C-termini were described as characteristic for bHLH-PAS class I proteins [15]. All the disease-associated missense mutations are located within the long C-terminus (Figure 3(Ac), Supplementary Materials). Prediction of the LLPS propensity resulted in local maxima in the linker region between the bHLH and PAS domains, the linker region between the PAS1 and PAS2 domains, and in the N-terminal region of the C-terminus (residue 390). The segment between residues 350–400 deserves special attention. It is predicted not only as highly disordered and possessing a local maximum of the LLPS propensity, but is also enriched in the PTM sites. What is more, many disease-associated mutations are reported in this region. According to HuVarBase, SIM1 missense mutations are linked mainly to skin cancer (H394Y, H402Y, D424N, S428F, S454L, R471Q, R493C, R550C, P588L, S603F, P661L, and R665C). The other diseases are lung cancer (R192H, G392R, E530K, A570G, N650Y, and S701C), breast cancer (P352T and A494T), liver cancer (H559Q, G448C, and Q704H), large intestine cancer (L217P, A371V, C472W, R548Q, and S663L), stomach cancer (S541L), hematopoietic and lymphoid tissue cancer (G408R and T481M), CNS cancer (P539R), esophagus cancer (E725K), and Schaaf-Yang syndrome (Q704L) (Supplementary Materials).

As demonstrated [53], the SIM1 mutation located in the C-terminus (p.G715V) leads to a novel SIM1 variant presenting reduced transcriptional activity. An ab initio hybrid model generated by Blackburn et al. [53] localized the p.G715 residue to the long IDR, directly in a small helix that is facing towards the solvent. The discussed helix is determined in our predictions as a local minimum in the disorder profiles generated by all predictors used in this study (Figure 3(Ac)), which is surrounded by highly disordered regions. Such a result is characteristic for motifs acting as the molecular recognition elements/features (MoREs/MoRFs), representing short interaction-prone segments that can undergo disorder-to-order transition upon binding to specific partners [104]. The substitution of G to V at this position increases the local hydrophobicity and may affect helix function and stability. This mutation could alter affinities for cofactors binding, regulatory functions and proteins structure, which can modulate the SIM1 target gene regulation [53].

In the case of SIM2, most of the documented PTM sites (Figure 3(Ba)), similar to the predicted MoRFs (Figure 3(Bb)), are placed along the long, highly disordered C-terminus (residues 336–667) (Figure 3(Bc)). The only modification documented for this protein is phosphorylation. Similar to previously analyzed bHLH-PAS TFs, most of the missense, disease-associated mutations are observed within the long IDRs or short, local disordered regions (Figure 3(Bc)). Predicted LLPS propensity shows a local maximum in the linker between bHLH and PAS (residues 54–76), which is also predicted as disordered. Curiously, although this region does not possess experimentally determined PTM sites, NetPhos predictor [105] suggests many putative phosphorylation sites are located in this region (Supplementary Materials), which also contains a high number of missense mutations. According to the HuVarBase, SIM2 missense mutations are linked mainly to lung cancer (S343Y, S355F, P385H, T646P, and Q469P), skin cancer (P57S, M164I, E339K, E345K, M377I, P448S, D450N, and F454S), liver cancer (F56L, A70T, G174S, and F394S), and large intestine cancer (A63V, A169V, D202N, and T433M). The other mutation-associated diseases are endometrium cancer (K190N), cervix cancer (K368N), fallopian tube cancer (C489G), hematopoietic and lymphoid tissue cancer (A350S), bone cancer (S199Y), thyroid cancer (L483M), and upper aerodigestive tract cancer (S502W) (Supplementary Materials).

### 2.3. Hif-2α

**Figure 4:**
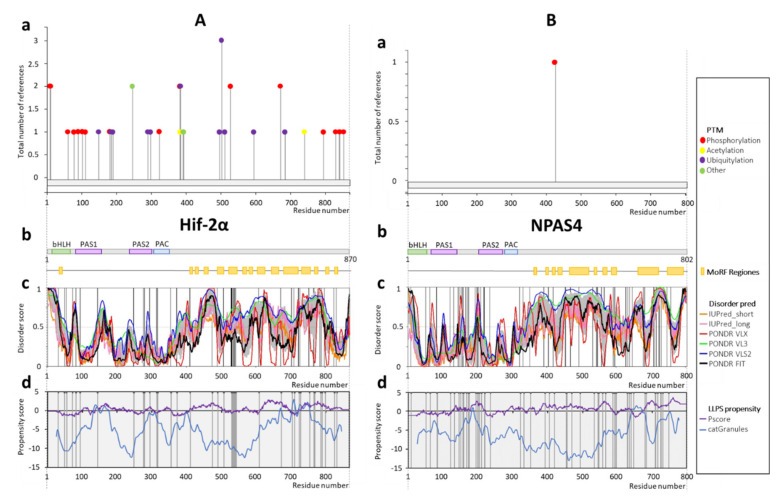
Schematic presentation of results for (**A**) Hif-2α (Q99814) and (**B**) NPAS4 (Q8IUM7) (**B**) analysis. (**a**) Post-translational modifications based on PhosphoSitePlus server [99]; (**b**) the domain structure of protein, green indicates the bHLH domain (14–47aa Hif-2α; 1–53aa NPAS4), purple represents PAS domains (84–154aa PAS1, 230–300aa PAS2 Hif-2α; 70–144aa PAS1, 203–273aa PAS2 NPAS4), whereas blue indicates PAC (304–347aa PAC Hif-2α; 278–317aa PAC NPAS4). Predicted MoRFs [100] are indicated as orange rectangles, (**c**) D^2^P^2^ database disorder regions predictions based on the protein amino acids sequence (find the legend in the plot for description). Grey shadow presents the averaged disorder profile, and a score over 0.5 indicates a high probability of disorder. Positions of disease-linked mutations are marked as black vertical lines (listed in HuVarBase database [101], Supplementary Materials), (**d**) LLPS propensity predictions based on catGranules (blue line) [102] and PScore (purple line) [103] servers; positions of disease-linked mutations are marked as black vertical lines (listed in HuVarBase database [101], Supplementary Materials).

For Hif-2α, most of the documented PTM sites (Figure 4(Aa)) and MoRFs (Figure 4(Ab)) are placed along the long C-terminus (residues 348–870) and within the linker between the bHLH and PAS1 domains (residues 48–83), both predicted as IDRs (Figure 4(Ac)). Similarly, most of the missense mutations in the Hif-2α sequence are located within the disordered C-terminus and the linker between the bHLH and PAS1 domains (residues 48–83) (Figure 4(Ab,c)). Interestingly, some of the Hif-2α documented PTMs are observed in the region comprising the PAS1 domain (see Figure 4(Aa,b)). This can be explained by the significantly higher local structural flexibility of regions surrounding this domain, in comparison to those of AhR or SIM proteins. Hif-2α is highly targeted by phosphorylation and ubiquitination, which can easily affect the life-time of the protein. Predicted LLPS profile contains many maxima throughout the entire protein length (Figure 4(Ad)). Importantly, these regions coincide with the predicted disordered fragments. Hif-2α missense mutations are mostly linked to familial erythrocytosis (A410T, M535V, M535T, G537R, G537W, F540L, F608L, S703A, T766P, P785T, I789V, R798G, R825Q, and E832D). The others mutation-associated diseases are autonomic ganglia cancer (L529P, A530T, A530E, and D539Y), large intestine cancer (S372N, Y489H, S672Y, and N768T), adrenal gland cancer (P531L, P531S, and Y532C), pancreas cancer (T776P and A530T), hematopoietic and lymphoid tissue cancer (E82K), ovary cancer (S723N), stomach cancer (S474T), prostate cancer (M507T), lung cancer (S72L), liver cancer (L542R), and esophagus cancer (D753E) (Supplementary Materials).

### 2.4. NPAS4

NPAS4 is one of the immediate early genes (IEGs) that can activate mechanisms related to the first defense against many cellular stresses [106]. Importantly, IEGs are regulated by a specific stimulus with no need for a de novo protein synthesis [107]. To date, there is only one documented NPAS4 modification—phosphorylation (Figure 4(Ba)) located in the bHLH domain, in the region where a locally disordered fragment of the sequence begins (between bHLH and PAS1 domains) (Figure 4(Bb,c)); however, NetPhos predictions showed many putative phosphorylation sites on the entire length of the protein (Supplementary Materials). Results of the disorder prediction indicated the presence of the long IDR in the C-terminal part of the protein (residues 318–802) and additional short IDRs within the N-terminal part of NPAS4, comprising bHLH and PAS domains, especially in the PAS1/PAS2 linker (residues 145–202) and less clearly in the bHLH/PAS1 linker (residues 54–69) (Figure 3(Bc)). Interestingly, the sites with high LLPS propensities (Figure 3(Bd)) mostly coincide with the IDRs. An exception is the central part of a protein (approximately residues 350–600) with a low LLPS potential and a high probability of being disordered. Similar to the protein sequences analyzed previously, disease-associated missense mutations of the NPAS4 sequence are located within IDRs, mostly predicted also as presenting a putative ability for LLPS formation. Especially interesting is the part of the C-terminus (residues 550–700) predicted as IDR with a high LLPS propensity which contains many described point mutations. NPAS4 missense mutations are linked predominantly to liver cancer (R150L, P194L, Q332K, P405L, Q547H, I639V, D647N, P679L, S683I, and S747F), skin cancer (R145C, P194S, D419N, L455F, P533S, P533L, S544N, T558I, D716N, E725K, and D730N), large intestine cancer (R159C, R172Q, P199H, L322I, and L351I) and esophagus cancer (A175T, A592V, and V710M). The other reported cancers associated with the NPAS4 mutations are upper aerodigestive tract (S453C and Q469H), breast (R200H and E628G), kidney (R595W), stomach (T708M), endometrium (P597S), thyroid (S493L), pancreas (R634H), cervix (Q629H), bone (E724K), and CNS (T587M) (Supplementary Materials).

### 2.5. ARNT2 and BMAL1

**Figure 5:**
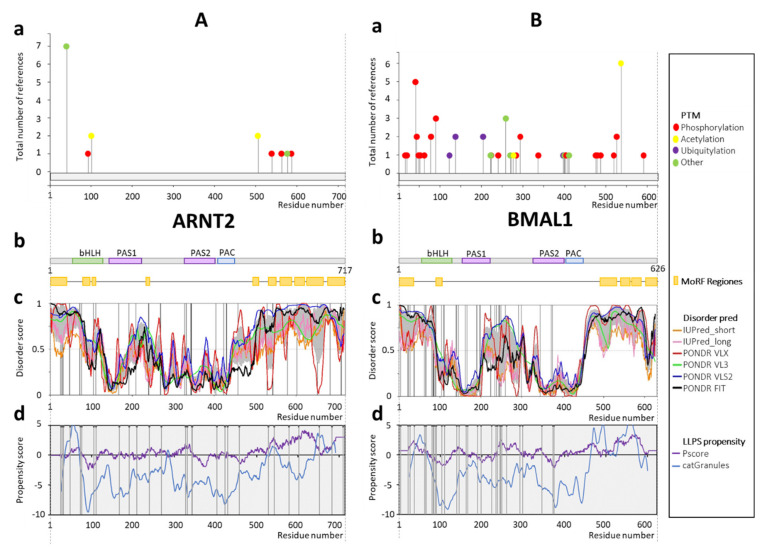
Schematic presentation of results for (**A**) ARNT2 (Q9HBZ2) and (**B**) BMAL1 (O00327) analysis. (**a**) Post-translation modifications based on PhosphoSitePlus server [99]; (**b**) the domain structure of protein, green indicates the bHLH domain (63–116aa ARNT2;72–125aa BMAL1); purple represents PAS domains (134–209aa PAS1, 323–393aa PAS2, ARNT2; 143–215aa PAS1, 326–396aa PAS2 BMAL1), whereas blue indicates PAC (398–441aa PAC ARNT2; 401–444aa PAC BMAL1). Predicted MoRFs [100] are indicated as orange rectangles, (**c**) D^2^P^2^ database disorder regions predictions based on the protein amino acids sequence (find the legend in the plot for description). Grey shadow presents the averaged disorder profile, and a score over 0.5 indicates a high probability of disorder. Positions of disease-linked mutations are marked as black vertical lines (listed in HuVarBase database [101], Supplementary Materials), (**d**) LLPS propensity predictions based on catGranules (blue line) [102] and PScore (purple line) [103] servers; positions of disease-linked mutations are marked as black vertical lines (listed in HuVarBase database [101], Supplementary Materials).

To compare different classes of bHLH-PAS TFs, we conducted analysis similar to that previously described for class I proteins, for ARNT2 and BMAL1—two representatives of the class II bHLH-PAS proteins. For ARNT2, documented PTMs (Figure 5(Aa)) and MoRFs (Figure 5(Ab)) are located within the N- and C-terminal regions predicted as highly disordered (Figure 5(Ac)). However, predicted phosphorylation sites are uniformly distributed along the protein (Supplementary Materials). The long, predicted as highly disordered linker between PAS1 and PAS2 domains (Figure 5(Ab,c)) contains short MoRFs (see Figure 5(Ab)). The high structural flexibility of the central part of this protein, which is much higher in comparison with the previously described class I members, could explain the ability of class II proteins to serve as an interaction partner for different class I proteins. Most of the missense mutations in the protein sequence are located within the C-terminus and within other regions predicted as disordered (Figure 5(Ac)). Prediction of the LLPS propensity generated many maxima spread over the entire protein length (Figure 5(Ad)). This seems to be a characteristic property of the class II bHLH-PAS TFs. Again, LLPS positive regions overlap with the disordered fragments. ARNT2 disease-associated missense variants are linked to large intestine cancer (A28V, R47C, R240K, P579S, and T602M), skin cancer (S458L and P529S), CNS cancer (Y430N), lung cancer (A25T and V683L), liver cancer (D191G and G710A), hematopoietic and lymphoid tissue cancer (H543R), pancreas cancer (P269S) and stomach cancer (G31R) (Supplementary Materials).

In the case of BMAL1 almost all documented PTM sites (Figure 5(Ba)) are distributed along the long C-terminus (residues 445–626), N-terminus (residues 1–71), and the linker between PAS1 and PAS2 domains (residues 216–325). However, similar to several other bHLH-PAS TFs, NetPhos predicts many phosphorylation sites uniformly distributed along the protein (Supplementary Materials). Predicted MoRFs occur also within the N- and C-terminal regions of BMAL1 (Figure 5(Bb)). All these fragments are predicted as highly disordered (Figure 5(Bc)). Importantly, the long disordered region in the middle part of BMAL1, characteristic of the class II factors, is observed (Figure 5(Bc)). For both BMAL1 and ARNT2, MoRFs were predicted within the N-terminal region (Figure 5(Bb)). All these features distinguish class II proteins and suggest their specific characteristics that allow them to interact with a wide spectrum of partners from the class I. In contrast to all previously analyzed bHLH-PAS proteins, no disease-associated missense mutation was reported in the disordered C-terminal region of BMAL1. Instead, missense mutations accumulated in the disordered N-terminal part (Figure 5(Bc)). This was unexpected, since the C-terminal TAD plays important roles in the mammalian clock regulation [94]. Importantly, acetylation of BMAL1 K537 was shown to be indispensable for circadian rhythmicity [108], suggesting the possibility that not all mutations responsible for disease development are known. LLPS propensity analysis revealed the presence of potential regions capable of phase separation in the N- and C-termini in accordance with the IDR prediction. BMAL1 seems to have a wider spectrum of PTMs (phosphorylation, ubiquitination, acetylation, and SUMOylation) in comparison to ARNT2. BMAL1 disease-associated missense mutations are linked predominantly to large intestine cancer (D22N, S27Y, R37C, R37H, R244Q, and V260A). The other related diseases are esophagus cancer (E62Q), genital tract cancer (E65K), thyroid cancer (H66P and C249R), skin cancer (P234H), cervix cancer (S246C), pancreas cancer (P292T), stomach cancer (T224S), breast cancer (T140S), and liver cancer (Q4L) (Supplementary Materials).

**Figure 6:**
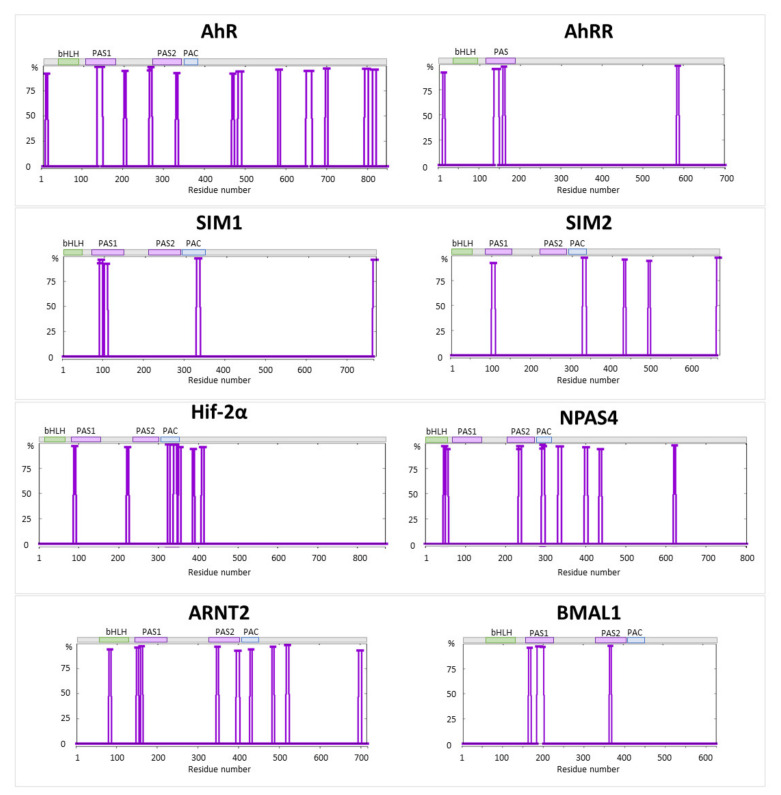
In silico prediction of amylogenic regions for AhR, AhRR, SIM1, SIM2, Hif-2α, NPAS4, ARNT2, and BMAL1 using Waltz predictor [109].

Finally, we evaluated the presence of the amylogenic regions in selected bHLH-PAS TFs (Figure 6). Our analysis revealed that all of the selected proteins were predicted to contain short amylogenic regions. Interestingly, most of these regions were located in N- and C-terminal regions of the defined domains, presenting higher flexibility. These regions show local N-terminal increase/C-terminal decrease of predicted disorder score in the corresponding intrinsic disorder profiles (see Figure 2, Figure 3, Figure 4 and Figure 5).

## 3. Discussion

Functional analysis of proteins at the crossroads between the different signaling pathways and, simultaneously, interacting with multiple partners (hub proteins), has proven that the intrinsically disordered nature of the interacting regions is indispensable [23]. Additionally, the DNA-binding proteins in eukaryotes were shown to be significantly enriched in disordered domains [110]. As aforementioned, bHLH-PAS proteins act as essential TFs via their binding to DNA and interacting with many physiological partners.

The results of our analysis confirm a high intrinsic disorder content of the bHLH-PAS TFs, especially in their long C-terminal regions. Additionally, short IDRs located in the region preceding the bHLH domain and in the linker between PAS domains can also be distinguished.

Utilizing the HuVarBase data in combination with the in silico analysis of selected representatives of the bHLH-PAS family allowed us to show that missense mutations associated with diseases are located mostly within predicted IDRs. For most of the analyzed proteins (AhRR, SIM1, Hif-2α, and NPAS4), we also predicted high propensities for LLPS in their putative IDRs. Furthermore, predicted mutations are often located at or in close proximity to the residues undergoing PTMs (Table 1).

By analyzing the presented data, we have noticed some mutation patterns (Table 1). Very often serine, a residue susceptible to phosphorylation, was substituted by a residue that is devoid of hydroxyl group, thereby unable be targeted to undergo such PTMs, for example: AHR/S733F, AHRR/S53G, SIM1/S3L, SIM1/S680L, Hif-2α/S703A, NPAS4/S683I ARNT2/S332L or BMAL1/S90I. On the contrary, often some residues predicted as involved in LLPS were substituted by serine, for example: AHR/P838S, AHRR/P283S SIM1/G271S, SIM2/P57S, Hif-2α/P531S, NPAS4/P194S, and ARNT2/P423S. These observations suggest that the peculiarities of the protein PTM pattern, especially within its IDR regions, is important for disease development.

We also observed that the G/A substitution (for example, SIM1/A570G and ARNT2/G710A) could influence the folding propensity of the corresponding region, since glycine is a known helix-breaker, whereas alanine favors α-helix formation. Some mutations could obviously change the physico-chemical properties of a polypeptide chain. For example, E/K substitution causes the change of the sign of the amino acid residue charge (for example: AHR/E488K, SIM1/E155K, SIM2/E106K, Hif-2α/E82K, NPAS4/E724K, ARNT2/E72K or BMAL1/E65K). In other cases, however, for example for R/K (AHR/R554K, ARNT2/R240K) or L/I/V (AHR/V570I, AHRR/I226V, SIM1/V326I, SIM2/V76I, SIM2/L283V, NPAS4/I639V, ARNT2/V110I, BMAL1/V162I), substitution impact was not so obvious, though such substitution also resulted in a deleterious effect. An example would be the K537R mutation of BMAL1, which prevented acetylation of this protein and resulted in inhibition of transcriptional repression important for the rhythmicity of circadian clock [108]. Another example is given by the V304I mutation of the bHLH-PAS family member, NPAS3. In fact, V304I was identified as an NPAS3 missense variant associated with psychiatric disorders. Although the V304I mutation located in the PAS linker did not alter the protein’s molecular function, mutation in the disordered region of NPAS3 led to the aggregation of this protein, which resulted in schizophrenia [111,112]. This has led us to hypothesize that some mutations could impact IDRs, thus promoting their misfolding and aggregation. Amyloid structures are widespread in nature for beneficial purposes, such as the formation of functional amyloids. However, misfolding and aggregation can lead to the formation of toxic amyloids often associated with the appearance of aberrant interactions of oligomeric intermediates with endogenous cellular components [113] resulting in disease development. Interestingly, although some proteins containing long IDRs were shown to have a propensity toward aggregate formation, it was also proposed that this aggregation tendency could be due to the aggregation-prone properties of the structured regions of the aggregating proteins [114]. In line with recent studies [115], we hypothesize that, in some cases, mutations could lead to the enhanced protein aggregation by modulating the exposure of the aggregation-prone regions.

**Figure 7:**
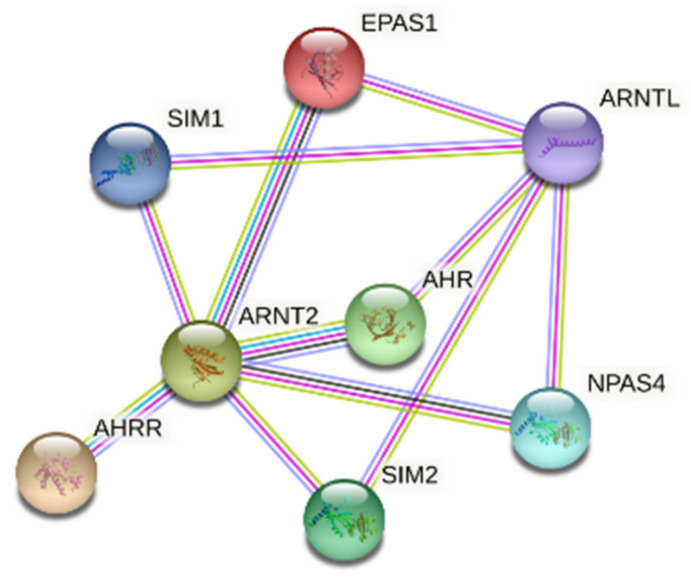
STRING-based interactome between selected representatives of bHLH-PAS transcription factor (TF) proteins (an internal protein-protein interaction network (PPI)). In the corresponding STRING-generated network, the nodes correspond to proteins, whereas the edges show predicted or known functional associations. Seven types of evidence are used to build the corresponding network, where they are indicated by the differently colored lines: a green line represents neighborhood evidence; a red line—the presence of fusion evidence; a purple line—experimental evidence; a blue line—co-occurrence evidence; a light blue line—database evidence; a yellow line—text mining evidence; and a black line—co-expression evidence.

Functionalities of IDPs and proteins containing IDRs usually rely on their abilities to interact with other proteins to form complexes and finally to organize PPI networks. This ensures the connection of different signaling pathways and promotes the creation of larger networks [116]. Protein interactivity can be evaluated using a publicly available computational platform STRING, which integrates all the information on PPIs, complements it with computational predictions and returns a PPI network showing all possible PPIs of a query protein(s) [117]. STRING-generated visualization of the internal interactome of selected bHLH-PAS members is presented in Figure 7. In line with earlier studies, Figure 7 shows that the bHLH-PAS proteins can interact with each other forming a rather well-linked PPI network.

**Figure 8:**
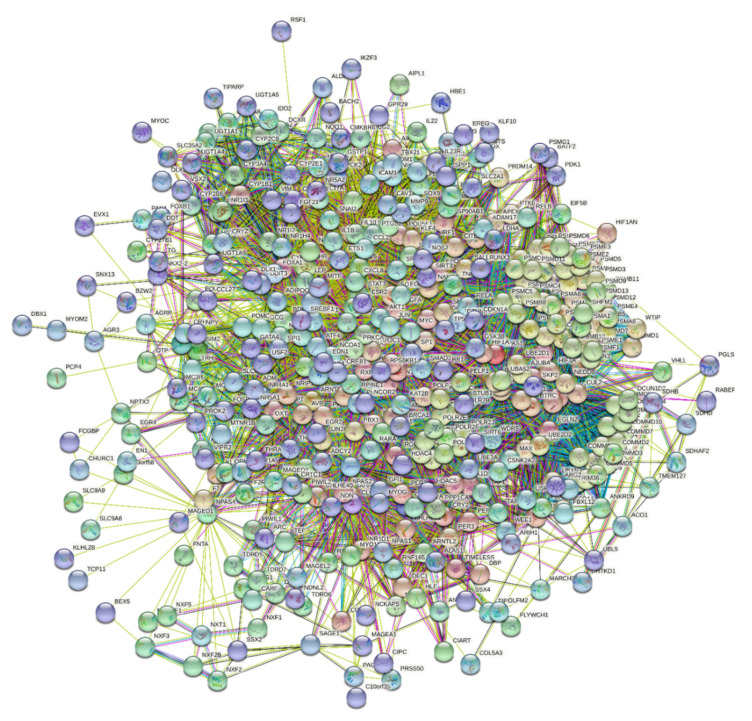
STRING-based external interactome of selected bHLH-PAS TFs with the “first shell” interactors. A confidence level of 0.5 was used in this analysis.

Since bHLH-PAS TFs usually function as hub proteins at the intersections of many signaling pathways, a high binding promiscuity is extremely important for their activities. Therefore, we used STRING to study the engagement of the bHLH-PAS TFs in interactions with the proteins forming the first shell of the resulting interactome. In this analysis, a confidence level of 0.5 was used. Figure 8 represents the resulting interactome that includes 432 nodes (proteins) connected by 8235 edges (interactions between proteins). Therefore, this interactome is characterized by an average node degree of 38.1 and shows an average local clustering coefficient of 0.589. Here, the average local clustering coefficient is a measure that defines how close neighbors of a given network are to forming a complete clique (i.e., a network, where each node, also known in graph theory as a vertex, is adjacent to each other vertex in the network). Therefore, the local clustering coefficient is equal to 1 if every neighbor connected to a given node *N_i_* is also connected to every other node within the neighborhood, and it is equal to 0 if no node that is connected to a given node *N_i_* connects to any other node that is connected to *N_i_*. The expected number of interactions for the set of proteins of the network of this size is 3516 indicating that this PPI network centered at the bHLH-PAS TFs has significantly more interactions than expected (PPI enrichment *p*-value is <10^−16^). Here, PPI enrichment *p*-value is a reflection of the fact that query proteins in the analyzed PPI network have more interactions among themselves than what would be expected for a random set of proteins of similar size, drawn from the genome. It was pointed out that such an enrichment indicates that the proteins are at least partially biologically connected, as a group.

We also used STRING to investigate the interactivity of individual bHLH-PAS TFs. The corresponding results are presented in the Supplementary Materials and clearly illustrate that all these TFs are promiscuous binders interacting with large numbers of specific partners.

The functionalities of IDPs and IDRs may depend on the abilities of such regions to undergo a disorder to order transition after binding [118]. Disease-associated missense mutations were most often found in PPI-controlling regions [119], known as MoRFs [34]. This indicates that pathogenesis may be associated with the wrong MoRF conformation after a missense mutation occurs. Recently, it was shown that the transition of the peptide mimicking a MoRF to a conformation with pronounced α-helical structure could be distorted by an amino acid substitution with proline as a helix breaker [120]. Activities of MoRFs responsible for PPI or protein localization are also regulated by PTMs, which may induce protein conformational changes. If so, the missense mutations of the residues serving as PTM targets can serve as important sites involved in disease induction after substitution [121].

The activities of bHLH-PAS TFs depend on nucleocytoplasmic shuttling, occurring as the result of interactions with proteins responsible for nuclear export/import. Nuclear localization signal (NLS) or nuclear export signal (NES) sequences were defined in the bHLH and PAS domains as well as in the C-terminal unstructured region of AhR. C-termini of Hif-1α and Hif-2α also contain conserved NLS and NES sequences. For SIM2 the C-terminal region cytoplasmic localization was documented [122]. Finally, we have previously demonstrated the presence of overlapping NES and NLS in the C-terminal region of NPAS4 [123]. PTMs, such as phosphorylation, especially those taking place in close proximity to the NLS/, were shown to regulate the intracellular distribution of proteins via activation/deactivation of the localization motifs [124]. This suggests that the disease-associated missense mutations located in the C-termini of bHLH-PAS TFs could affect the NLS/NES activities by substitutions of residues in a signal sequence itself, or by substitutions of residues located close to the signal sequence that are important for this signal’s activity.

It was shown that cells organize many biochemical processes in specific compartments known as MLOs originating as a result of LLPS. In the nucleus, LLPS is responsible for formation of nucleoli, paraspeckles, and Cajal bodies created by factors regulating, among other processes, chromatin remodeling, transcription, and RNA processing. Such LLPS-driven MLOs can serve as rapid recyclers/reactive storage facilities, which supply or sequester TFs [125]. Altered phase separation affects the disassembly of protein condensates, resulting in their accumulation, which could lead to pathological processes [126]. Interestingly, LLPS of a disease-causing mutant of heterogeneous nuclear ribonucleoprotein A1 (hnRNPA1, D262V) was shown to promote fibrillization of this protein, whereas MLO containing the wild type protein did not [127]. Pathological neurodegeneration related to age or disease and protein aggregation have been also linked to LLPS-driven processes [26]. Proteins containing long IDRs represent an abundant class of macromolecules that can phase separately under physiological conditions. IDRs do not have stable 3D structures and often contain repeated sequence elements providing the basis for multivalent weakly adhesive intermolecular interactions responsible for LLPS formation [128]. Recently, we discussed bHLH TFs as factors putatively engaged in the formation of LLPS during transcription process [31]. We propose that the aberrant regulation of LLPS processes by disease-associated bHLH-PAS variants with specific missense mutations could result in disease development. Obviously, computational results reported in our study require experimental validation. However, they generate testable hypotheses, and therefore these data provide an important foundation for future studies dedicated to the analysis of the effects of mutations in ordered regions, on conformational changes affecting PPIs and the propensities to make LLPS.

## 4. Materials and Methods

We have used UniProt (https://www.uniprot.org/, (accessed on 11 March 2021)) as a freely accessible resource of protein sequences. We have used canonical sequences of human proteins: AhR (UniProtKB—P35869), AhRR (UniProtKB—A9YTQ3), SIM1 (UniProtKB—P81133), SIM2 (UniProtKB—Q14190), Hif-2α (UniProtKB—Q99814), NPAS4 (UniProtKB—Q8IUM7), ARNT (UniProtKB—P27540) and BMAL1 (UniProtKB—O00327) as our research objects.

To search disease-associated mutations, we have reviewed the literature and analyzed the Human Variants Database (HuVarBase) https://www.iitm.ac.in/bioinfo/huvarbase/mas18srch.php, (accessed on 11 March 2021) [101]. HuVarBase is a comprehensive database on human genome variants reported in the databases, such as Humsavar (Human polymorphisms and disease mutations), 1000 Genomes (genetic variants occurring at least in 1% of studied populations), SwissVar (portal to search variants in Swiss-Prot entries of the UniProt Knowledgebase), ClinVar (aggregates information about genomic variation and its relationship to human health), and COSMIC (the Catalogue Of Somatic Mutations In Cancer).

We performed in silico IDR and MoRF analyses using The Database of Disordered Protein Prediction (D^2^P^2^) platform [129] (http://d2p2.pro/, (accessed on 11 March 2021)), along with commonly used disorder predictors of the PONDR family, PONDR^®^ VLXT [130], PONDR^®^ VL3 [131], PONDR^®^ VLS2 [132], and PONDR^®^ FIT [133], as well as IUPred2A (Short) and IUPred2A (Long) [134,135]. These predictors were selected based on their specific features. PONDR^®^ VLXT is sensitive to local sequence peculiarities [130]; PONDR^®^ VSL2 is one of the more accurate stand-alone disorder predictors [132,136,137]; whereas PONDR^®^ VL3 possesses high accuracy in finding long IDRs [131]. PONDR-FIT [133] is a meta-predictor combining six individual predictors, PONDR^®^ VLXT [130], PONDR^®^ VL3 [131], PONDR^®^ VLS2 [132], FondIndex [138], IUPred [134], and TopIDP [139]. This meta-predictor is slightly more accurate than its individual components and other predictors. Finally, IUPred2A provides evaluations of short and long disordered regions [134,135].

Many IDPs and IDRs include disorder-based interaction motifs such as molecular recognition features (MoRFs) [104,140,141,142] that can undergo binding-induced folding and are utilized by IDPs/IDRs in formation of various complexes and assemblages. Such disorder-based binding sites were predicted by an ANCHOR algorithm [100].

Additionally, we performed computational analyses of the predisposition of query proteins to undergo LLPS using catGranule [102] (http://service.tartaglialab.com/update_submission/216885/dd56e32a89, (accessed on 11 March 2021)) and PScore [103] (http://abragam.med.utoronto.ca/~JFKlab/Software/psp.htm, (accessed on 11 March 2021)) servers.

We used the PhophoSitePlus database (https://www.phosphosite.org/homeAction, (accessed on 11 March 2021)) to take a look at the known experimentally documented PTM sites [99], and Waltz predictor (trained on a large set of experimentally characterized amyloid forming peptides) for detection of putative amylogenic regions in proteins [109] (https://waltz.switchlab.org/, (accessed on 11 March 2021)). Settings used for Waltz prediction were “Best Overall Performance” and pH 7.0.

We evaluated protein interactivity using a publicly available computational platform STRING (https://string-db.org/, (accessed on 11 March 2021)) which is an online database that integrates a variety of types of information on protein-protein interactions (PPIs), and complements this with computational predictions and produces a PPI network showing all possible PPIs based on a query protein(s) [117].

We performed predictions of phosphorylation sites using the NetPhos 3.1 server, (http://www.cbs.dtu.dk/services/NetPhos/, (accessed on 11 March 2021)) [105].

## 5. Conclusions

In this study, we conducted extensive analyses of the presence of IDRs and LLPS propensities combined with the analyses of human polymorphism and PTM databases, and the results have led us to conclude that most of the disease-associated missense mutations occur in IDRs of analyzed bHLH-PAS family members, which are located in close proximity to the regions important for LLPS regulation, or susceptible to PTMs. Changes in the PTM patterns can affect protein interaction network, protein stability or protein shuttling regulation. Importantly, mutations can also impact propensities for protein aggregation. All such variations can modify protein functions and induce specific disease states. Unfortunately, to date few experimental studies have been conducted concerning the structural characterization of bHLH-PAS IDRs and LLPS of these proteins. This can be explained by difficulties with the expression of proteins containing long IDRs. In the current study, we used available in silico predictors and databases to summarize the current state of knowledge. However, a better understanding of structure and function dependency cannot be achieved without in vivo and/or in vitro experimental data. Therefore, we emphasize the need for conducting further experimental research in these directions, as one of the most importantly future tasks that can enable us to open new perspectives and to gain a better understanding of the roles of LLPS and IDRs in bHLH-PAS TF functioning and development of various diseases.

## Figures and Tables

**Table 1:** Summary of AHR, AHRR, SIM1/2, Hif-2α, NPAS4, ARNT2 and BMAL1 mutations, disorder scores, and PTM and LLPS analyses. Protein mutations (based on HuVarBase) are arranged in order. Disorder scores are determined by mean predicted intrinsic disorder score (PIDS_mean_). Ordered regions (PIDS_mean_ ≤ 0.15), flexible (i.e., with 0.15 < PIDS_mean_ ≤ 0.5), and disordered (PIDS_mean_ ≥ 0.5) regions are indicated by blue, pink, and red colors, respectively. Closely located documented PTMs (PhosphoSitePlus, distance < 12aa) are listed. PTM sites coinciding with mutation sites are highlighted in yellow. Abbreviations: ac—acetylation, m—methylation, p—phosphorylation, sm—sumoylation, ub—ubiquitylation. Predicted LLPS is marked with ‘+’, ‘+local’ for local maxima of predicted LLPS and ‘++’ for global maximum. Residues predicted as disordered, with close mutation sites and LLPS positive score are highlighted in gray.

No.	Gene Name	Protein Mutation	Disorder Score	Close Post = Translational Modifications (PTMs)	LLPS
1	AHR	P18L	0.81 ± 0.17	S12p, K17ac, K24ac,ub,sm	
2	AHR	D132N	0.03 ± 0.03		
3	AHR	T141N	0.08 ± 0.06		+
4	AHR	Q150K	0.14 ± 0.10		+
5	AHR	E169K	0.20 ± 0.09		+local
6	AHR	T199P	0.43 ± 0.19		+
7	AHR	P260L	0.24 ± 0.11	K254sm	
8	AHR	N284H	0.15 ± 0.08	K292ub	+
9	AHR	R305K	0.12 ± 0.06		+
10	AHR	T311I	0.18 ± 0.10	Y322p	
11	AHR	R368C	0.22 ± 0.15		+
12	AHR	Q383H	0.39 ± 0.18	T387p	
13	AHR	R398Q	0.45 ± 0.10		+
14	AHR	E488K	0.48 ± 0.17		++
15	AHR	N505S	0.51 ± 0.10	K510ub	++
16	AHR	T507I	0.55 ± 0.14	K510ub	++
17	AHR	R554K	0.24 ± 0.08	K560ub	+
18	AHR	V570I	0.24 ± 0.09	K560ub	
19	AHR	S733F	0.58 ± 0.15		
20	AHR	P838S	0.69 ± 0.07		+
**No.**	**Gene Name**	**Protein Mutation**	**Disorder Score**	**Close PTMs**	**LLPS**
1	AHRR	V29M	0.90 ± 0.07	K24ub	+
2	AHRR	S53G	0.37 ± 0.19		
3	AHRR	S63F	0.22 ± 0.19		+
4	AHRR	Q88R	0.44 ± 0.22		+
5	AHRR	A96V	0.72 ± 0.10		+
6	AHRR	P102S	0.76 ± 0.14		+
7	AHRR	A112V	0.45 ± 0.15		+
8	AHRR	T152M	0.08 ± 0.08		+
9	AHRR	P189A	0.43 ± 0.16		
10	AHRR	I226V	0.04 ± 0.05		+local
11	AHRR	R230C	0.05 ± 0.06		
12	AHRR	P283S	0.45 ± 0.22	S281p	+
13	AHRR	R285W	0.52 ± 0.20	S281p	+
14	AHRR	A300T	0.63 ± 0.21	K322ub	+
15	AHRR	A301V	0.53 ± 0.18	K322ub	+
16	AHRR	A371T	0.77 ± 0.07	K371ub	++
17	AHRR	T419I	0.92 ± 0.03	K402ub	
18	AHRR	G427E	0.92 ± 0.06		
19	AHRR	R485W	0.65 ± 0.26		
20	AHRR	R491W	0.66 ± 0.28		
21	AHRR	R491Q	0.66 ± 0.28		
22	AHRR	G494S	0.63 ± 0.27		
23	AHRR	T524M	0.57 ± 0.10	K538sm	
24	AHRR	C545F	0.43 ± 0.10	K538sm	+
25	AHRR	V553M	0.30 ± 0.12	K577sm	+
26	AHRR	G612S	0.49 ± 0.22	T605p	
27	AHRR	D645H	0.54 ± 0.24	R643m	+
28	AHRR	A674S	0.68 ± 0.13	K660ub,sm	
**No.**	**Gene Name**	**Protein Mutation**	**Disorder Score**	**Close PTMs**	**LLPS**
1	SIM1	E3D	0.88 ± 0.13		+local
2	SIM1	R10W	0.81 ± 0.13		
3	SIM1	S31L	0.28 ± 0.10	S31p	+
4	SIM1	Q36P	0.27 ± 0.12		+
5	SIM1	G65D	0.36 ± 0.13		
6	SIM1	D74Y	0.40 ± 0.17		+local
7	SIM1	E155K	0.13 ± 0.10		+
8	SIM1	R192H	0.08 ± 0.08	K181ac	+
9	SIM1	R192C	0.08 ± 0.08	K181ac	+
10	SIM1	V213M	0.17 ± 0.12		
11	SIM1	L217P	0.23 ± 0.17		
12	SIM1	V222I	0.22 ± 0.14		+local
13	SIM1	E224K	0.19 ± 0.13		+local
14	SIM1	A236T	0.06 ± 0.06		
15	SIM1	H268Q	0.08 ± 0.06		+
16	SIM1	H268Y	0.08 ± 0.06		+
17	SIM1	G271S	0.08 ± 0.07		+
18	SIM1	T292N	0.07 ± 0.05		+local
19	SIM1	G303S	0.03 ± 0.02		+
20	SIM1	S309G	0.10 ± 0.06		
21	SIM1	A311V	0.12 ± 0.07		+local
22	SIM1	V326I	0.17 ± 0.10	S343p	+
23	SIM1	P352T	0.47 ± 0.12	S343p, S350p, S355p, Y356p, S358p	+
24	SIM1	A371V	0.84 ± 0.14	S378p	++
25	SIM1	G392R	0.73 ± 0.14	S382p	+local
26	SIM1	H394Y	0.71 ± 0.16	S382p	+
27	SIM1	E396D	0.67 ± 0.19	S382p	+
28	SIM1	E399K	0.68 ± 0.21	S382p	
29	SIM1	H402Y	0.73 ± 0.16		+local
30	SIM1	G408R	0.81 ± 0.09		
31	SIM1	D424N	0.75 ± 0.10		+
32	SIM1	S428F	0.63 ± 0.16		+
33	SIM1	A432T	0.56 ± 0.20		+
34	SIM1	A435T	0.49 ± 0.19		+
35	SIM1	G448C	0.28 ± 0.14		
36	SIM1	S454L	0.28 ± 0.14		+
37	SIM1	R471Q	0.28 ± 0.10	Y477p	
38	SIM1	C472W	0.28 ± 0.11	Y477p, T481p	
39	SIM1	T481M	0.32 ± 0.12	T481p	+local
40	SIM1	R493C	0.43 ± 0.10		
41	SIM1	A494T	0.43 ± 0.09		
42	SIM1	E530K	0.66 ± 0.18		
43	SIM1	P539R	0.83 ± 0.07		+local
44	SIM1	S541L	0.83 ± 0.08		
45	SIM1	R548Q	0.85 ± 0.06		
46	SIM1	R550C	0.84 ± 0.06		
47	SIM1	H559Q	0.78 ± 0.12		+local
48	SIM1	A570G	0.77 ± 0.09		+
49	SIM1	P588L	0.69 ± 0.09		
50	SIM1	S603F	0.36 ± 0.18		
51	SIM1	N650Y	0.75 ± 0.12	S642p, S651p	
52	SIM1	R657W	0.76 ± 0.13	S651p, S660p	+
53	SIM1	P661L	0.75 ± 0.13	S660p, S670p	+local
54	SIM1	S663L	0.70 ± 0.19	S660p, S670p	+local
55	SIM1	R665C	0.67 ± 0.17	S660p, S670p	
56	SIM1	S680L	0.43 ± 0.16	S670p	
57	SIM1	S701C	0.22 ± 0.13		
58	SIM1	Q704H	0.16 ± 0.11		
59	SIM1	Q704L	0.16 ± 0.11		
60	SIM1	E725K	0.17 ± 0.11		+
**No.**	**Gene Name**	**Protein Mutation**	**Disorder Score**	**Close PTMs**	**LLLPS**
1	SIM2	A40V	0.22 ± 0.08		+
2	SIM2	R44G	0.15 ± 0.05		+
3	SIM2	F56L	0.15 ± 0.07		++
4	SIM2	P57S	0.16 ± 0.08		++
5	SIM2	A63V	0.29 ± 0.10		+
6	SIM2	A70T	0.33 ± 0.13		+
7	SIM2	V76I	0.36 ± 0.14		+local
8	SIM2	V92F	0.05 ± 0.02		
9	SIM2	E106K	0.17 ± 0.06	S115p	
10	SIM2	A108T	0.18 ± 0.08	S115p	
11	SIM2	T120M	0.18 ± 0.09	S115p	
12	SIM2	I124M	0.23 ± 0.06	S115p	
13	SIM2	Y125H	0.27 ± 0.07	S115p	
14	SIM2	D134N	0.28 ± 0.12		+
15	SIM2	P145L	0.24 ± 0.09		+local
16	SIM2	H147Y	0.24 ± 0.10		+local
17	SIM2	M164I	0.08 ± 0.06		+
18	SIM2	L168F	0.08 ± 0.06		+
19	SIM2	A169V	0.09 ± 0.06		++
20	SIM2	G174S	0.09 ± 0.07	Y188p	
21	SIM2	K190N	0.05 ± 0.05	Y188p	
22	SIM2	Y194H	0.05 ± 0.05	Y188p	
23	SIM2	S199Y	0.05 ± 0.06	Y188p	+
24	SIM2	D202N	0.04 ± 0.04		+
25	SIM2	V211G	0.14 ± 0.15		+local
26	SIM2	A212V	0.15 ± 0.17	Y228p, S229p	+
27	SIM2	A221T	0.19 ± 0.13	Y228p, S229p	
28	SIM2	T223I	0.16 ± 0.10	Y228p, S229p	+
29	SIM2	M231I	0.05 ± 0.04	Y228p, S229p	+local
30	SIM2	D239Y	0.05 ± 0.04	S237p	+
31	SIM2	L240P	0.05 ± 0.04	S237p	+
32	SIM2	D246N	0.10 ± 0.07	S237p	+
33	SIM2	T253M	0.22 ± 0.15		+
34	SIM2	G254R	0.24 ± 0.13		++
35	SIM2	E262K	0.15 ± 0.13		+
36	SIM2	H267Y	0.07 ± 0.05		+
37	SIM2	G271D	0.06 ± 0.05		++
38	SIM2	D273N	0.07 ± 0.06		+
39	SIM2	R278C	0.04 ± 0.04		
40	SIM2	A280T	0.04 ± 0.03		
41	SIM2	L283V	0.06 ± 0.04		+local
42	SIM2	G303S	0.06 ± 0.04		
43	SIM2	A311V	0.17 ± 0.14		
44	SIM2	V313A	0.23 ± 0.13		
45	SIM2	R318L	0.31 ± 0.20		
46	SIM2	R318H	0.31 ± 0.20		
47	SIM2	C324Y	0.21 ± 0.07		
48	SIM2	C324F	0.21 ± 0.07		
49	SIM2	V326M	0.17 ± 0.06		
50	SIM2	V326G	0.17 ± 0.06		
51	SIM2	E339K	0.23 ± 0.12	S343p	+
52	SIM2	S343Y	0.33 ± 0.12	S343p	+
53	SIM2	E345K	0.37 ± 0.12	S343p, S348p, T349p	+
54	SIM2	A350S	0.41 ± 0.18	S348p, T349p, A350p, S352p	+
55	SIM2	S355F	0.54 ± 0.11	S348p, T349p, A350p, S352p, 3T58p	+
56	SIM2	K368N	0.79 ± 0.15	T358p, T366p	+
57	SIM2	M377I	0.78 ± 0.14	T366p	
58	SIM2	P385H	0.61 ± 0.16	T383p	
59	SIM2	F394S	0.51 ± 0.24	T383p	+
60	SIM2	T433M	0.44 ± 0.20		+
61	SIM2	P448S	0.33 ± 0.23		+
62	SIM2	D450N	0.35 ± 0.22		
63	SIM2	F454S	0.38 ± 0.21		
64	SIM2	Q469P	0.32 ± 0.17	S471p	
65	SIM2	L483M	0.28 ± 0.22		
66	SIM2	C489G	0.30 ± 0.22		+local
67	SIM2	S502W	0.78 ± 0.12		
68	SIM2	S503Y	0.79 ± 0.13		
69	SIM2	T646P	0.78 ± 0.13		+
**No.**	**Gene Name**	**Protein Mutation**	**Disorder Score**	**Close PTMs**	**LLPS**
1	Hif-2α	T31M	0.60 ± 0.33		
2	Hif-2α	S49Y	0.39 ± 0.13		+local
3	Hif-2α	S55F	0.25 ± 0.17	S62p, S79p	+
4	Hif-2α	S72L	0.44 ± 0.11	S62p, S79p	+
5	Hif-2α	E82K	0.54 ± 0.18	S79p, Y91p	+
6	Hif-2α	A94T	0.16 ± 0.06	Y91p, T103p	
7	Hif-2α	R144C	0.47 ± 0.13	K150ub	+
8	Hif-2α	H248N	0.23 ± 0.15	R247m	+local
9	Hif-2α	S276L	0.17 ± 0.07		++
10	Hif-2α	E279V	0.19 ± 0.06		+
11	Hif-2α	Q294H	0.29 ± 0.16	K291ub	
12	Hif-2α	G314E	0.11 ± 0.08	T324p	
13	Hif-2α	V317M	0.06 ± 0.04	T324p	
14	Hif-2α	S355F	0.29 ± 0.07		
15	Hif-2α	S372N	0.28 ± 0.16	S383p, K385ac	++
16	Hif-2α	A410T	0.45 ± 0.10	K392ub, K394sm	+
17	Hif-2α	S474T	0.84 ± 0.13		+local
18	Hif-2α	Y489H	0.56 ± 0.18	K497ub	+local
19	Hif-2α	M507T	0.40 ± 0.12	K497ub	+local
20	Hif-2α	L529P	0.55 ± 0.12	T528p	
21	Hif-2α	A530V	0.52 ± 0.14	T528p	
22	Hif-2α	A530T	0.52 ± 0.14	T528p	
23	Hif-2α	A530E	0.52 ± 0.14	T528p	
24	Hif-2α	P531L	0.53 ± 0.14	T528p	+
25	Hif-2α	P531S	0.53 ± 0.14	T528p	+
26	Hif-2α	Y532C	0.54 ± 0.13	T528p	+local
27	Hif-2α	M535V	0.58 ± 0.12	T528p	
28	Hif-2α	M535T	0.58 ± 0.12	T528p	
29	Hif-2α	G537R	0.56 ± 0.13	T528p	
30	Hif-2α	G537W	0.56 ± 0.13	T528p	
31	Hif-2α	D539Y	0.58 ± 0.12	T528p	
32	Hif-2α	F540L	0.60 ± 0.10		
33	Hif-2α	L542R	0.56 ± 0.18		
34	Hif-2α	F608L	0.60 ± 0.19	K595ub	+local
35	Hif-2α	S672Y	0.57 ± 0.15	S672p	+
36	Hif-2α	S703A	0.43 ± 0.15		+
37	Hif-2α	R710Q	0.40 ± 0.16		+
38	Hif-2α	S723N	0.69 ± 0.08		++
39	Hif-2α	P727L	0.64 ± 0.10	K741ac	+
40	Hif-2α	D753E	0.68 ± 0.12	K741ac	+local
41	Hif-2α	T766P	0.64 ± 0.25		
42	Hif-2α	N768T	0.65 ± 0.28		
43	Hif-2α	P785T	0.84 ± 0.08		+local
44	Hif-2α	I789V	0.80 ± 0.10	S795p	
45	Hif-2α	R798G	0.58 ± 0.13	S795p	
46	Hif-2α	R825Q	0.33 ± 0.20	S830p	
47	Hif-2α	E832D	0.27 ± 0.11	S830p, T840p	
**No.**	**Gene Name**	**Protein Mutation**	**Disorder Score**	**Close PTMs**	**LLPS**
1	NPAS4	A8T	0.70 ± 0.17		
2	NPAS4	R51H	0. 05 ± 0.03		+local
3	NPAS4	A63V	0.24 ± 0.13		
4	NPAS4	P82H	0.12 ± 0.10		+local
5	NPAS4	G83S	0.12 ± 0.09		+
6	NPAS4	D121N	0.13 ± 0.06		
7	NPAS4	R132H	0.21 ± 0.08		+
8	NPAS4	R145C	0.28 ± 0.09		
9	NPAS4	R150L	0.36 ± 0.20		+
10	NPAS4	S156F	0.43 ± 0.19		+
11	NPAS4	R159C	0.39 ± 0.18		++
12	NPAS4	V167M	0.24 ± 0.07		+
13	NPAS4	R172Q	0.16 ± 0.09		+local
14	NPAS4	A175T	0.16 ± 0.11		
15	NPAS4	P194S	0.41 ± 0.13		+
16	NPAS4	P194L	0.41 ± 0.13		+
17	NPAS4	P199H	0.67 ± 0.07		+local
18	NPAS4	R200H	0.67 ± 0.08		+local
19	NPAS4	G204D	0.65 ± 0.16		+
20	NPAS4	A210V	0.40 ± 0.10		
21	NPAS4	S219N	0.18 ± 0.15		
22	NPAS4	R220H	0.16 ± 0.15		+local
23	NPAS4	I236V	0.10 ± 0.10		
24	NPAS4	L322I	0.35 ± 0.10		+
25	NPAS4	Q332K	0.43 ± 0.12		
26	NPAS4	L351I	0.59 ± 0.13		+local
27	NPAS4	R392Q	0.65 ± 0.21		+local
28	NPAS4	P405L	0.63 ± 0.16		
29	NPAS4	D419N	0.60 ± 0.16	T423p, T427p	
30	NPAS4	S453C	0.80 ± 0.10		+local
31	NPAS4	L455F	0.83 ± 0.09		
32	NPAS4	Q469H	0.71 ± 0.19		
33	NPAS4	S493L	0.80 ± 0.10		
34	NPAS4	P533S	0.79 ± 0.11		+
35	NPAS4	P533L	0.79 ± 0.11		+
36	NPAS4	S544N	0.71 ± 0.15		
37	NPAS4	Q547H	0.75 ± 0.12		+
38	NPAS4	T558I	0.60 ± 0.22		+
39	NPAS4	T587M	0.56 ± 0.13		
40	NPAS4	G566E	0.48 ± 0.24		
41	NPAS4	A592V	0.36 ± 0.15		+
42	NPAS4	R595W	0.35 ± 0.16		+
43	NPAS4	P597S	0.41 ± 0.13		+
44	NPAS4	E628G	0.43 ± 0.11		++
45	NPAS4	Q629H	0.42 ± 0.11		++
46	NPAS4	R634H	0.47 ± 0.13		++
47	NPAS4	I639V	0.49 ± 0.11		++
48	NPAS4	D647N	0.59 ± 0.07		++
49	NPAS4	P679L	0.54 ± 0.14		
50	NPAS4	S683I	0.41 ± 0.16		+
51	NPAS4	T708M	0.71 ± 0.13		+
52	NPAS4	V710M	0.73 ± 0.13		+
53	NPAS4	D716N	0.85 ± 0.09		+
54	NPAS4	E724K	0.92 ± 0.07		+
55	NPAS4	E725K	0.94 ± 0.06		+
56	NPAS4	D730N	0.95 ± 0.05		+
57	NPAS4	S747F	0.76 ± 0.08		+
**No.**	**Gene Name**	**Protein Mutation**	**Disorder Score**	**Close PTMs**	**LLPS**
1	ARNT2	A25T	0.76 ± 0.18		+
2	ARNT2	A28V	0.76 ± 0.19		++
3	ARNT2	G31R	0.78 ± 0.18		++
4	ARNT2	R47C	0.78 ± 0.14	R42m	+
5	ARNT2	E72K	0.78 ± 0.14		
6	ARNT2	R76W	0.71 ± 0.12		+local
7	ARNT2	I105V	0.47 ± 0.13	S94p, K102ac	
8	ARNT2	V110I	0.51 ± 0.16	K102ac	+
9	ARNT2	V167I	0.30 ± 0.14		
10	ARNT2	D191G	0.44 ± 0.09		+local
11	ARNT2	R209Q	0.48 ± 0.15		
12	ARNT2	R240K	0.37 ± 0.20		+local
13	ARNT2	P269S	0.41 ± 0.12		+
14	ARNT2	M328I	0.36 ± 0.13		+local
15	ARNT2	S332L	0.30 ± 0.06		+
16	ARNT2	S343F	0.22 ± 0.08		+local
17	ARNT2	D344N	0.21 ± 0.08		+local
18	ARNT2	D344G	0.21 ± 0.08		+local
19	ARNT2	R404C	0.18 ± 0.13		
20	ARNT2	P423S	0.15 ± 0.11		+local
21	ARNT2	Y430N	0.15 ± 0.08		+
22	ARNT2	S458L	0.52 ± 0.15		+
23	ARNT2	P529S	0.62 ± 0.21	S540p	+
24	ARNT2	H543R	0.58 ± 0.21	S540p	+
25	ARNT2	P579S	0.80 ± 0.11	R578m, S588p	+local
26	ARNT2	T602M	0.84 ± 0.11	S588p	+local
27	ARNT2	R652Q	0.65 ± 0.28		
28	ARNT2	V683L	0.87 ± 0.04		
29	ARNT2	G710A	0.63 ± 0.16		
**No.**	**Gene Name**	**Protein Mutation**	**Disorder Score**	**Close PTMs**	**LLPS**
1	BMAL1	Q4L	0.88 ± 0.12		
2	BMAL1	D22N	0.74 ± 0.15	S17p	++
3	BMAL1	S27Y	0.74 ± 0.14	S17p, T21p	++
4	BMAL1	R37C	0.76 ± 0.14	S42p, T44p	+
5	BMAL1	R37H	0.76 ± 0.14	S42p, T44p	+
6	BMAL1	E62Q	0.78 ± 0.08	T52p, Y63p	+local
7	BMAL1	E65K	0.77 ± 0.07	Y63p	
8	BMAL1	H66P	0.76 ± 0.07	Y63p	
9	BMAL1	I80F	0.76 ± 0.11	S78p, S90p	+local
10	BMAL1	R83Q	0.69 ± 0.13	S78p, S90p	+local
11	BMAL1	R84H	0.68 ± 0.11	S78p, S90p	+local
12	BMAL1	R85Q	0.65 ± 0.14	S78p, S90p	+local
13	BMAL1	M88I	0.58 ± 0.15	S78p, S90p	
14	BMAL1	S90I	0.50 ± 0.12	S90p	
15	BMAL1	A104T	0.29 ± 0.10		+
16	BMAL1	D110Y	0.30 ± 0.07		+
17	BMAL1	T140S	0.23 ± 0.13	K138ub	+
18	BMAL1	D145N	0.17 ± 0.12	K138ub	+
19	BMAL1	D145E	0.17 ± 0.12	K138ub	+
20	BMAL1	V162I	0.03 ± 0.02		+
21	BMAL1	R166G	0.05 ± 0.03		+
22	BMAL1	Q190E	0.123 ± 0.08		
23	BMAL1	P198L	0.19 ± 0.10	K205ub	+local
24	BMAL1	T224S	0.58 ± 0.18	K223ub, T224p	+
25	BMAL1	P234H	0.54 ± 0.17	K223ub, T224p	
26	BMAL1	R238Q	0.49 ± 0.21	S241p	+local
27	BMAL1	R244Q	0.47 ± 0.24	S241p	
28	BMAL1	S246C	0.48 ± 0.24	S241p	
29	BMAL1	C249R	0.50 ± 0.29	S241p, K259sm	
30	BMAL1	V260A	0.60 ± 0.22	K259sm	+
31	BMAL1	P292T	0.41 ± 0.16	T294p	+
32	BMAL1	D299Y	0.58 ± 0.06	T294p	+
33	BMAL1	A345T	0.12 ± 0.05	S337p	
34	BMAL1	S372L	0.08 ± 0.08		+
35	BMAL1	E375G	0.08 ± 0.06		+

## Supplementary Materials

The following are available online at https://www.mdpi.com/1422-0067/22/6/2868/s1, Supplementary Materials (pdf file containing results of HuVarBase analysis and STRING plots for individual proteins).
