# Supplementary material for: The Participation of the Intrinsically Disordered Regions of the bHLH-PAS Transcription Factors in Disease Development
Source: Int J Mol Sci. 2021 Mar 11;22(6):2868. doi: 10.3390/ijms22062868 (PMC8001110; doi:10.3390/ijms22062868)
Supplement: Supplementary file 1 [file ijms-22-02868-s001.pdf]

The participation of the intrinsically disordered regions of the bHLH-PAS transcription factors in disease development

Marta Kolonko-Adamska [1], Vladimir N. Uversky [2,3], Beata Greb-Markiewicz [1,*]

[1] Department of Biochemistry, Molecular Biology and Biotechnology, Faculty of Chemistry, Wroclaw University of Science and Technology, Wybrzeze Wyspianskiego 27, 50-370 Wroclaw, Poland
[2] Department of Molecular Medicine, USF Health Byrd Alzheimer's Research Institute, Morsani College of Medicine, University of South Florida, Tampa, FL, United States
[3] Laboratory of New Methods in Biology, Institute for Biological Instrumentation, Russian Academy of Sciences, Federal Research Center "Pushchino Scientific Center for Biological Research of the Russian Academy of Sciences", Pushchino, Moscow region, 142290 Russia
* Correspondence: beata.greb-markiewicz@pwr.edu.pl; Tel.: 0048713206226

Supplementary Materials

1) **Results of the HuVarBase analysis of hAHR, hAHRR, hSIM1, hSIM2, hHIF2a, hNPAS4, hARNT2, and hBMAL1.**
2) **Results of the NetPhos 3.1 server phosphorylation sites prediction for hAHR, hAHRR, hSIM1, hSIM2, hHIF2a, hNPAS4, hARNT2, and hBMAL1.**
3) **STRING-based external interactome of selected bHLH-PAS TFs with the "first shell" interactors. The confidence level of 0.7 was used in this analysis.**
4) **Plots of STRING analysis of individual proteins: hAHR, hAHRR, hSIM1, hSIM2, hHIF2a, hNPAS4, hARNT2, and hBMAL1.**

1) Results of the analysis of hAHR, hAHRR, hSIM1, hSIM2, hHIF2a, hNPAS4, hARNT2, and hBMAL1 by HuVarBase, Human Variants Database: https://www.iitm.ac.in/bioinfo/huvarbase/mas18srch.php

Mutations taking place in ordered (i.e., with the predicted intrinsic disorder scores evaluated by mean disorder predictor, ordered ($PIDS_{mean}$, ≤ 0.15), flexible (i.e., with 0.15 < $PIDS_{mean}$ ≤ 0.5), and disordered regions $PIDS_{mean}$ ≥ 0.5) are indicated by blue, pink, and red characters, respectively. Numeric values of the corresponding disorder scores are shown in the brackets.

hAHR (UniProt P35869)

No.	Gene Name	Protein Mutation	Disease / Tissue (COSMIC)	Database
1	AHR	P18L (0.81±0.17)	liver, cancer	COSMIC
2	AHR	D132N (0.034±0.029)	lung, cancer	COSMIC
3	AHR	T141N (0.079±0.055)	central_nervous_system, cancer	COSMIC
4	AHR	Q150K (0.135±0.096)	liver, cancer	COSMIC
5	AHR	E169K (0.20±0.09)	breast, cancer	COSMIC
6	AHR	T199P (0.43±0.19)	large_intestine, cancer	COSMIC
7	AHR	P260L (0.24±0.11)	large_intestine, cancer	COSMIC
8	AHR	N284H (0.149±0.077)	large_intestine, cancer	COSMIC
9	AHR	R305K (0.121±0.061)	large_intestine, cancer	COSMIC
10	AHR	T311I (0.176±0.096)	skin, cancer	COSMIC
11	AHR	R368C (0.22±0.15)	stomach, cancer	COSMIC
12	AHR	Q383H (0.39±0.18)	oesophagus, cancer	COSMIC
13	AHR	R398Q (0.45±0.10)	large_intestine, cancer	COSMIC
14	AHR	E488K (0.48±0.17)	kidney, cancer	COSMIC
15	AHR	N505S (0.508±0.098)	large_intestine, cancer	COSMIC
16	AHR	T507I (0.55±0.14)	large_intestine, cancer	COSMIC
17	AHR	R554K (0.243±0.078)	soft_tissue, cancer	COSMIC<br>Humsavar
18	AHR	V570I (0.244±0.093)	thyroid, cancer	COSMIC<br>Humsavar
19	AHR	S733F (0.58±0.15)	liver, cancer	COSMIC
20	AHR	P838S (0.688±0.070)	large_intestine, cancer	COSMIC

hAHRR (UniProt A9YTQ3)

No.	Gene Name	Protein Mutation	Disease / Tissue (COSMIC)	Database
1	AHRR	V29M (0.898±0.066)	large_intestine, cancer	COSMIC
2	AHRR	S53G (0.37±0.19)	stomach, cancer	COSMIC
3	AHRR	S63F (0.22±0.19)	large_intestine, cancer	COSMIC
4	AHRR	Q88R (0.44±0.22)	large_intestine, cancer	COSMIC
5	AHRR	A96V (0.72±0.10)	large_intestine, cancer	COSMIC
6	AHRR	P102S (0.76±0.14)	large_intestine, cancer	COSMIC
7	AHRR	A112V (0.45±0.15)	liver, cancer	COSMIC
8	AHRR	T152M (0.083±0.075)	large_intestine, cancer	COSMIC
9	AHRR	P189A (0.43±0.16)	central_nervous_system, cancer	COSMIC<br>Humsavar
10	AHRR	I226V (0.042±0.046)	large_intestine, cancer	COSMIC
11	AHRR	R230C (0.053±0.055)	large_intestine, cancer	COSMIC
12	AHRR	P283S (0.45±0.22)	skin, cancer	COSMIC
13	AHRR	R285W (0.52±0.20)	large_intestine, cancer	COSMIC
14	AHRR	A300T (0.63±0.21)	large_intestine, cancer	COSMIC
15	AHRR	A301V (0.53±0.18)	skin, cancer	COSMIC
16	AHRR	A371T (0.773±0.070)	endometrium, cancer	COSMIC
17	AHRR	T419I (0.919±0.028)	large_intestine, cancer	COSMIC
18	AHRR	G427E (0.924±0.063)	skin, cancer	COSMIC
19	AHRR	R485W (0.65±0.26)	large_intestine, cancer	COSMIC
20	AHRR	R491W (0.66±0.28)	large_intestine, cancer	COSMIC
21	AHRR	R491Q (0.66±0.28)	prostate, cancer	ClinVar<br>COSMIC
22	AHRR	G494S (0.63±0.27)	large_intestine, cancer	COSMIC
23	AHRR	T524M (0.565±0.098)	large_intestine, cancer	COSMIC
24	AHRR	C545F (0.433±0.099)	liver, cancer	COSMIC
25	AHRR	V553M (0.30±0.12)	large_intestine, cancer	COSMIC
26	AHRR	G612S (0.49±0.22)	oesophagus, cancer	COSMIC
27	AHRR	D645H (0.54±0.24)	prostate, cancer	COSMIC
28	AHRR	A674S (0.68±0.13)	liver, cancer	COSMIC

hSIM1 (UniProt P81133)

No.	Gene Name	Protein Mutation	Disease / Tissue (COSMIC)	Database
1	SIM1	E3D (0.88±0.13)	large_intestine, cancer	COSMIC
2	SIM1	R10W (0.81±0.13)	large_intestine, cancer	COSMIC
3	SIM1	S31L (0.28±0.10)	urinary_tract, cancer	COSMIC
4	SIM1	Q36P (0.27±0.12)	lung, cancer	COSMIC
5	SIM1	G65D (0.36±0.13)	upper_aerodigestive_tract, cancer	COSMIC
6	SIM1	D74Y (0.40±0.17)	urinary_tract, cancer	COSMIC
7	SIM1	E155K (0.13±0.10)	skin, cancer	COSMIC
8	SIM1	R192H (0.081±0.083)	lung, cancer	COSMIC
9	SIM1	R192C (0.081±0.083)	large_intestine, cancer	COSMIC
10	SIM1	V213M (0.17±0.12)	cervix, cancer	COSMIC
11	SIM1	L217P (0.23±0.17)	large_intestine, cancer	COSMIC
12	SIM1	V222I (0.22±0.14)	stomach, cancer	COSMIC
13	SIM1	E224K (0.19±0.13)	skin, cancer	COSMIC
14	SIM1	A236T (0.064±0.060)	large_intestine, cancer	COSMIC
15	SIM1	H268Q (0.084±0.055)	Schaaf-yang syndrome	ClinVar
16	SIM1	H268Y (0.084±0.055)	skin, cancer	COSMIC
17	SIM1	G271S (0.084±0.071)	oesophagus, cancer	COSMIC
18	SIM1	T292N (0.071±0.051)	liver, cancer	COSMIC
19	SIM1	G303S (0.027±0.023)	stomach, cancer	COSMIC
20	SIM1	S309G (0.095±0.059)	upper_aerodigestive_tract, cancer	COSMIC
21	SIM1	A311V (0.119±0.067)	large_intestine, cancer	COSMIC
22	SIM1	V326I (0.17±0.10)	stomach, cancer	COSMIC
23	SIM1	P352T (0.47±0.12)	breast, cancer	1000 Genomes (data @ Ensembl) ClinVar COSMIC Humsavar
24	SIM1	A371V (0.84±0.14)	large_intestine, cancer	1000 Genomes (data @ Ensembl) ClinVar COSMIC Humsavar
25	SIM1	G392R (0.73±0.14)	lung, cancer	COSMIC
26	SIM1	H394Y (0.71±0.16)	skin, cancer	COSMIC
27	SIM1	E396D (0.67±0.19)	large_intestine, cancer	COSMIC
28	SIM1	E399K (0.68±0.21)	skin, cancer	COSMIC
29	SIM1	H402Y (0.73±0.16)	skin, cancer	COSMIC
30	SIM1	G408R (0.809±0.089)	haematopoietic_and_lymphoid_tissue, cancer	COSMIC

31	SIM1	D424N (0.746±0.095)	skin, cancer	COSMIC
32	SIM1	S428F (0.63±0.16)	skin, cancer	COSMIC
33	SIM1	A432T (0.56±0.20)	large_intestine, cancer	COSMIC
34	SIM1	A435T (0.49±0.19)	stomach, cancer	COSMIC
35	SIM1	G448C (0.28±0.14)	liver, cancer	COSMIC
36	SIM1	S454L (0.28±0.14)	skin, cancer	COSMIC
37	SIM1	R471Q (0.28±0.10)	skin, cancer	COSMIC
38	SIM1	C472W (0.28±0.11)	large_intestine, cancer	COSMIC
39	SIM1	T481M (0.32±0.12)	haematopoietic_and_lymphoid_tissue, cancer	COSMIC
40	SIM1	R493C (0.43±0.10)	skin, cancer	COSMIC
41	SIM1	A494T (0.433±0.091)	breast, cancer	COSMIC
42	SIM1	E530K (0.66±0.18)	lung, cancer	COSMIC
43	SIM1	P539R (0.834±0.069)	central_nervous_system, cancer	COSMIC
44	SIM1	S541L (0.833±0.079)	stomach, cancer	COSMIC
45	SIM1	R548Q (0.846±0.059)	large_intestine, cancer	COSMIC
46	SIM1	R550C (0.835±0.063)	skin, cancer	COSMIC
47	SIM1	H559Q (0.78±0.12)	liver, cancer	COSMIC
48	SIM1	A570G (0.765±0.092)	lung, cancer	COSMIC
49	SIM1	P588L (0.688±0.092)	skin, cancer	COSMIC
50	SIM1	S603F (0.36±0.18)	skin, cancer	COSMIC
51	SIM1	N650Y (0.75±0.12)	lung, cancer	COSMIC
52	SIM1	R657W (0.76±0.13)	large_intestine, cancer	COSMIC
53	SIM1	P661L (0.75±0.13)	skin, cancer	COSMIC
54	SIM1	S663L (0.70±0.19)	large_intestine, cancer	COSMIC
55	SIM1	R665C (0.67±0.17)	skin, cancer	COSMIC
56	SIM1	S680L (0.43±0.16)	prostate, cancer	COSMIC
57	SIM1	S701C (0.22±0.13)	lung, cancer	COSMIC
58	SIM1	Q704H (0.16±0.11)	liver, cancer	COSMIC
59	SIM1	Q704L (0.16±0.11)	Schaaf-yang syndrome	ClinVar
60	SIM1	E725K (0.17±0.11)	oesophagus, cancer	ClinVar<br>COSMIC

hSIM2 (UniProt Q14190)

No.	Gene Name	Protein Mutation	Disease / Tissue (COSMIC)	Database
1	SIM2	A40V (0.215±0.080)	lung, cancer	COSMIC
2	SIM2	R44G (0.154±0.046)	kidney, cancer	COSMIC
3	SIM2	F56L (0.152±0.066)	liver, cancer	COSMIC
4	SIM2	P57S (0.163±0.079)	skin, cancer	COSMIC
5	SIM2	A63V (0.29±0.10)	large_intestine, cancer	COSMIC
6	SIM2	A70T (0.33±0.13)	liver, cancer	COSMIC
7	SIM2	V76I (0.36±0.14)	thyroid, cancer	COSMIC
8	SIM2	V92F (0.045±0.020)	skin, cancer	COSMIC
9	SIM2	E106K (0.167±0.064)	large_intestine, cancer	COSMIC
10	SIM2	A108T (0.181±0.075)	liver, cancer	COSMIC
11	SIM2	T120M (0.178±0.091)	central_nervous_system, cancer	COSMIC
12	SIM2	I124M (0.229±0.055)	stomach, cancer	COSMIC
13	SIM2	Y125H (0.273±0.074)	liver, cancer	COSMIC
14	SIM2	D134N (0.28±0.12)	lung, cancer	COSMIC
15	SIM2	P145L (0.240±0.090)	prostate, cancer	COSMIC
16	SIM2	H147Y (0.24±0.10)	thyroid, cancer	COSMIC
17	SIM2	M164I (0.083±0.059)	skin, cancer	COSMIC
18	SIM2	L168F (0.079±0.057)	large_intestine, cancer	COSMIC
19	SIM2	A169V (0.089±0.062)	large_intestine, cancer	COSMIC
20	SIM2	G174S (0.094±0.066)	liver, cancer	COSMIC
21	SIM2	K190N (0.046±0.049)	endometrium, cancer	COSMIC
22	SIM2	Y194H (0.053±0.054)	skin, cancer	COSMIC
23	SIM2	S199Y (0.052±0.057)	bone, cancer	COSMIC
24	SIM2	D202N (0.043±0.042)	large_intestine, cancer	COSMIC
25	SIM2	V211G (0.14±0.15)	breast, cancer	COSMIC
26	SIM2	A212V (0.15±0.17)	large_intestine, cancer	COSMIC
27	SIM2	A221T (0.19±0.13)	stomach, cancer	COSMIC
28	SIM2	T223I (0.16±0.10)	skin, cancer	COSMIC
29	SIM2	M231I (0.050±0.040)	liver, cancer	COSMIC
30	SIM2	D239Y (0.050±0.039)	liver, cancer	COSMIC
31	SIM2	L240P (0.053±0.039)	thyroid, cancer	COSMIC
32	SIM2	D246N (0.101±0.065)	skin, cancer	COSMIC
33	SIM2	T253M (0.22±0.15)	breast, cancer	COSMIC
34	SIM2	G254R (0.24±0.13)	skin, cancer	COSMIC
35	SIM2	E262K (0.15±0.13)	skin, cancer	COSMIC
36	SIM2	H267Y (0.070±0.045)	skin, cancer	COSMIC

37	SIM2	G271D (0.063±0.053)	large_intestine, cancer	COSMIC
38	SIM2	D273N (0.065±0.062)	skin, cancer	COSMIC
39	SIM2	R278C (0.043±0.035)	liver, cancer	COSMIC
40	SIM2	A280T (0.040±0.031)	large_intestine, cancer	COSMIC
41	SIM2	L283V (0.056±0.036)	prostate, cancer	COSMIC
42	SIM2	G303S (0.061±0.043)	pancreas, cancer	COSMIC
43	SIM2	A311V (0.17±0.14)	bone, cancer	COSMIC
44	SIM2	V313A (0.23±0.13)	lung, cancer	COSMIC
45	SIM2	R318L (0.31±0.20)	oesophagus, cancer	COSMIC
46	SIM2	R318H (0.31±0.20)	prostate, cancer	COSMIC
47	SIM2	C324Y (0.214±0.065)	thyroid, cancer	COSMIC
48	SIM2	C324F (0.214±0.065)	liver, cancer	COSMIC
49	SIM2	V326M (0.174±0.057)	large_intestine, cancer	COSMIC
50	SIM2	V326G (0.174±0.057)	thyroid, cancer	COSMIC
51	SIM2	E339K (0.23±0.12)	skin, cancer	COSMIC
52	SIM2	S343Y (0.33±0.12)	lung, cancer	COSMIC
53	SIM2	E345K (0.37±0.12)	skin, cancer	COSMIC
54	SIM2	A350S (0.41±0.18)	haematopoietic_and_lymphoid_tissue, cancer	COSMIC
55	SIM2	S355F (0.54±0.11)	lung, cancer	COSMIC
56	SIM2	K368N (0.79±0.15)	cervix, cancer	COSMIC
57	SIM2	M377I (0.78±0.14)	skin, cancer	COSMIC
58	SIM2	P385H (0.61±0.16)	lung, cancer	COSMIC
59	SIM2	F394S (0.51±0.24)	liver, cancer	COSMIC
60	SIM2	T433M (0.44±0.20)	large_intestine, cancer	COSMIC
61	SIM2	P448S (0.33±0.23)	skin, cancer	COSMIC
62	SIM2	D450N (0.35±0.22)	skin, cancer	COSMIC
63	SIM2	F454S (0.38±0.21)	skin, cancer	COSMIC
64	SIM2	Q469P (0.32±0.17)	lung, cancer	COSMIC
65	SIM2	L483M (0.28±0.22)	thyroid, cancer	COSMIC Humsavar
66	SIM2	C489G (0.30±0.22)	fallopian_tube, cancer	COSMIC
67	SIM2	S502W (0.78±0.12)	upper_aerodigestive_tract, cancer	COSMIC
68	SIM2	S503Y (0.79±0.13)	cervix, cancer	COSMIC
69	SIM2	T646P (0.78±0.13)	lung, cancer	COSMIC

hHIF-2α (EPAS1, UniProt Q99814)

No.	Gene Name	Protein Mutation	Disease / Tissue (COSMIC)	Database
1	EPAS1	T31M (0.60±0.33)	large_intestine, cancer	COSMIC
2	EPAS1	S49Y (0.39±0.13)	Familial erythrocytosis	ClinVar
3	EPAS1	S55F (0.25±0.17)	breast, cancer	COSMIC
4	EPAS1	S72L (0.44±0.11)	lung, cancer	COSMIC
5	EPAS1	E82K (0.54±0.18)	haematopoietic_and_lymphoid_tissue, cancer	COSMIC
6	EPAS1	A94T (0.155±0.062)	liver, cancer	COSMIC
7	EPAS1	R144C (0.47±0.13)	upper_aerodigestive_tract, cancer	COSMIC
8	EPAS1	H248N (0.23±0.15)	large_intestine, cancer	COSMIC
9	EPAS1	S276L (0.174±0.065)	large_intestine, cancer	COSMIC
10	EPAS1	E279V (0.190±0.060)	liver, cancer	COSMIC
11	EPAS1	Q294H (0.29±0.16)	Familial erythrocytosis	ClinVar
12	EPAS1	G314E (0.108±0.075)	haematopoietic_and_lymphoid_tissue, cancer	COSMIC
13	EPAS1	V317M (0.057±0.038)	large_intestine, cancer	COSMIC
14	EPAS1	S355F (0.290±0.072)	skin, cancer	COSMIC
15	EPAS1	S372N (0.28±0.16)	large_intestine, cancer	COSMIC
16	EPAS1	A410T (0.45±0.10)	Familial erythrocytosis	1000 Genomes (data @ Ensembl) ClinVar
17	EPAS1	S474T (0.84±0.13)	stomach, cancer	COSMIC
18	EPAS1	Y489H (0.56±0.18)	large_intestine, cancer	COSMIC
19	EPAS1	M507T (0.40±0.12)	prostate, cancer	COSMIC
20	EPAS1	L529P (0.55±0.12)	autonomic_ganglia, cancer	COSMIC
21	EPAS1	A530V (0.52±0.14)	autonomic_ganglia, cancer	COSMIC
22	EPAS1	A530T (0.52±0.14)	pancreas, cancer	COSMIC
23	EPAS1	A530E (0.52±0.14)	autonomic_ganglia, cancer	COSMIC
24	EPAS1	P531L (0.53±0.14)	adrenal_gland, cancer	COSMIC
25	EPAS1	P531S (0.53±0.14)	adrenal_gland, cancer	COSMIC
26	EPAS1	Y532C (0.54±0.13)	adrenal_gland, cancer	COSMIC
27	EPAS1	M535V (0.58±0.12)	erythrocytosis, familial, 4	ClinVar Humsavar SwissVar (variant page)
28	EPAS1	M535T (0.58±0.12)	erythrocytosis, familial, 4	Humsavar SwissVar (variant page)
29	EPAS1	G537R (0.56±0.13)	Erythrocytosis, familial, 4	ClinVar Humsavar SwissVar (variant page)

30	EPAS1	G537W (0.56±0.13)	Erythrocytosis, familial, 4	ClinVar<br>Humsavar<br>SwissVar<br>(variant page)
31	EPAS1	D539Y (0.58±0.12)	autonomic_ganglia, cancer	COSMIC
32	EPAS1	F540L (0.604±0.099)	erythrocytosis, familial, 4	Humsavar<br>SwissVar<br>(variant page)
33	EPAS1	L542R (0.56±0.18)	liver, cancer	COSMIC
34	EPAS1	F608L (0.60±0.19)	Familial erythrocytosis	ClinVar
35	EPAS1	S672Y (0.57±0.15)	large_intestine, cancer	COSMIC
36	EPAS1	S703A (0.43±0.15)	Familial erythrocytosis	ClinVar
37	EPAS1	R710Q (0.40±0.16)	lung, cancer	COSMIC
38	EPAS1	S723N (0.689±0.082)	ovary, cancer	COSMIC
39	EPAS1	P727L (0.635±0.095)	urinary_tract, cancer	COSMIC
40	EPAS1	D753E (0.68±0.12)	oesophagus, cancer	COSMIC
41	EPAS1	T766P (0.64±0.25)	pancreas, cancer	1000 Genomes<br>(data @ Ensembl)<br>ClinVar<br>COSMIC<br>Humsavar
42	EPAS1	N768T (0.65±0.28)	large_intestine, cancer	COSMIC
43	EPAS1	P785T (0.837±0.084)	Familial erythrocytosis	1000 Genomes<br>(data @ Ensembl)<br>ClinVar<br>Humsavar
44	EPAS1	I789V (0.80±0.10)	Familial erythrocytosis	ClinVar
45	EPAS1	R798G (0.58±0.13)	Familial erythrocytosis	1000 Genomes<br>(data @ Ensembl)<br>ClinVar
46	EPAS1	R825Q (0.328±0.096)	Familial erythrocytosis	ClinVar
47	EPAS1	E832D (0.27±0.11)	Familial erythrocytosis	1000 Genomes<br>(data @ Ensembl)<br>ClinVar

hNPAS4 (UniProt Q8IUM7)

No.	Gene Name	Protein Mutation	Disease / Tissue (COSMIC)	Database
1	NPAS4	A8T (0.70±0.17)	large_intestine, cancer	COSMIC
2	NPAS4	R51H (0. 047±0.031)	large_intestine, cancer	COSMIC
3	NPAS4	A63V (0.24±0.13)	stomach, cancer	COSMIC
4	NPAS4	P82H (0.121±0.099)	skin, cancer	COSMIC
5	NPAS4	G83S (0.115±0.094)	large_intestine, cancer	COSMIC
6	NPAS4	D121N (0.133±0.061)	large_intestine, cancer	COSMIC
7	NPAS4	R132H (0.210±0.083)	breast, cancer	COSMIC
8	NPAS4	R145C (0.277±0.090)	skin, cancer	COSMIC
9	NPAS4	R150L (0.36±0.20)	liver, cancer	COSMIC
10	NPAS4	S156F (0.43±0.19)	NS, cancer	COSMIC
11	NPAS4	R159C (0.39±0.18)	large_intestine, cancer	COSMIC
12	NPAS4	V167M (0.244±0.068)	breast, cancer	COSMIC
13	NPAS4	R172Q (0.157±0.091)	large_intestine, cancer	COSMIC
14	NPAS4	A175T (0.16±0.11)	oesophagus, cancer	COSMIC
15	NPAS4	P194S (0.41±0.13)	skin, cancer	COSMIC
16	NPAS4	P194L (0.41±0.13)	liver, cancer	COSMIC
17	NPAS4	P199H (0.669±0.070)	large_intestine, cancer	COSMIC
18	NPAS4	R200H (0.678±0.081)	breast, cancer	COSMIC
19	NPAS4	G204D (0.65±0.16)	central_nervous_system, cancer	COSMIC
20	NPAS4	A210V (0.40±0.10)	liver, cancer	COSMIC
21	NPAS4	S219N (0.18±0.15)	large_intestine, cancer	COSMIC
22	NPAS4	R220H (0.16±0.15)	large_intestine, cancer	COSMIC
23	NPAS4	I236V (0.10±0.10)	NS, cancer	COSMIC
24	NPAS4	L322I (0.35±0.10)	large_intestine, cancer	COSMIC
25	NPAS4	Q332K (0.43±0.12)	liver, cancer	COSMIC
26	NPAS4	L351I (0.59±0.13)	large_intestine, cancer	COSMIC
27	NPAS4	R392Q (0.65±0.21)	NS, cancer	COSMIC
28	NPAS4	P405L (0.63±0.16)	liver, cancer	COSMIC
29	NPAS4	D419N (0.60±0.16)	skin, cancer	COSMIC
30	NPAS4	S453C (0.803±0.098)	upper_aerodigestive_tract, cancer	COSMIC
31	NPAS4	L455F (0.831±0.089)	skin, cancer	COSMIC
32	NPAS4	Q469H (0.71±0.19)	upper_aerodigestive_tract, cancer	COSMIC
33	NPAS4	S493L (0.80±0.10)	thyroid, cancer	COSMIC
34	NPAS4	P533S (0.79±0.11)	skin, cancer	COSMIC
35	NPAS4	P533L (0.79±0.11)	skin, cancer	COSMIC
36	NPAS4	S544N (0.71±0.15)	skin, cancer	COSMIC
37	NPAS4	Q547H (0.75±0.12)	liver, cancer	COSMIC

38	NPAS4	T558I (0.60±0.22)	skin, cancer	COSMIC
39	NPAS4	T587M (0.56±0.13)	central_nervous_system, cancer	COSMIC Humsavar
40	NPAS4	G566E (0.48±0.24)	NS, cancer	COSMIC
41	NPAS4	A592V (0.36±0.15)	oesophagus, cancer	COSMIC
42	NPAS4	R595W (0.35±0.16)	kidney, cancer	COSMIC
43	NPAS4	P597S (0.41±0.13)	endometrium, cancer	COSMIC
44	NPAS4	E628G (0.43±0.11)	breast, cancer	COSMIC
45	NPAS4	Q629H (0.42±0.11)	cervix, cancer	COSMIC
46	NPAS4	R634H (0.47±0.13)	pancreas, cancer	COSMIC
47	NPAS4	I639V (0.49±0.11)	liver, cancer	COSMIC
48	NPAS4	D647N (0.585±0.068)	liver, cancer	COSMIC
49	NPAS4	P679L (0.54±0.14)	liver, cancer	COSMIC
50	NPAS4	S683I (0.41±0.16)	liver, cancer	COSMIC
51	NPAS4	T708M (0.71±0.13)	stomach, cancer	COSMIC
52	NPAS4	V710M (0.73±0.13)	oesophagus, cancer	COSMIC
53	NPAS4	D716N (0.846±0.088)	skin, cancer	COSMIC
54	NPAS4	E724K (0.915±0.073)	bone, cancer	COSMIC
55	NPAS4	E725K (0.937±0.059)	skin, cancer	COSMIC
56	NPAS4	D730N (0.951±0.052)	skin, cancer	COSMIC
57	NPAS4	S747F (0.759±0.079)	liver, cancer	COSMIC

hARNT2 (UniProt Q9HBZ2)

No.	Gene Name	Protein Mutation	Disease / Tissue (COSMIC)	Database
1	ARNT2	A25T (0.76±0.18)	lung, cancer	COSMIC
2	ARNT2	A28V (0.76±0.19)	large_intestine, cancer	COSMIC
3	ARNT2	G31R (0.78±0.18)	stomach, cancer	COSMIC
4	ARNT2	R47C (0.78±0.14)	large_intestine, cancer	COSMIC
5	ARNT2	E72K (0.78±0.14)	stomach, cancer	COSMIC
6	ARNT2	R76W (0.71±0.12)	endometrium, cancer	COSMIC
7	ARNT2	I105V (0.47±0.13)	central_nervous_system, cancer	COSMIC
8	ARNT2	V110I (0.51±0.16)	large_intestine, cancer	COSMIC
9	ARNT2	V167I (0.30±0.14)	liver, cancer	COSMIC
10	ARNT2	D191G (0.442±0.090)	liver, cancer	COSMIC
11	ARNT2	R209Q (0.48±0.15)	upper_aerodigestive_tract, cancer	COSMIC
12	ARNT2	R240K (0.37±0.20)	large_intestine, cancer	COSMIC
13	ARNT2	P269S (0.41±0.12)	pancreas, cancer	COSMIC
14	ARNT2	M328I (0.36±0.13)	lung, cancer	COSMIC
15	ARNT2	S332L (0.301±0.064)	pancreas, cancer	COSMIC
16	ARNT2	S343F (0.219±0.083)	upper_aerodigestive_tract, cancer	COSMIC
17	ARNT2	D344N (0.207±0.075)	large_intestine, cancer	COSMIC
18	ARNT2	D344G (0.207±0.075)	endometrium, cancer	COSMIC
19	ARNT2	R404C (0.18±0.13)	skin, cancer	COSMIC
20	ARNT2	P423S (0.15±0.11)	skin, cancer	COSMIC
21	ARNT2	Y430N (0.147±0.077)	central_nervous_system, cancer	COSMIC
22	ARNT2	S458L (0.52±0.15)	skin, cancer	COSMIC
23	ARNT2	P529S (0.62±0.21)	skin, cancer	COSMIC
24	ARNT2	H543R (0.58±0.21)	haematopoietic_and_lymphoid_tissue, cancer	COSMIC
25	ARNT2	P579S (0.80±0.11)	large_intestine, cancer	COSMIC
26	ARNT2	T602M (0.84±0.11)	large_intestine, cancer	COSMIC
27	ARNT2	R652Q (0.65±0.28)	large_intestine, cancer	COSMIC
28	ARNT2	V683L (0.868±0.040)	lung, cancer	COSMIC
29	ARNT2	G710A (0.63±0.16)	liver, cancer	COSMIC

hBMAL1 (UniProt O00327)

No.	Gene Name	Protein Mutation	Disease / Tissue (COSMIC)	Database
1	ARNTL	Q4L (0.88±0.12)	liver, cancer	COSMIC
2	ARNTL	D22N (0.74±0.15)	large_intestine, cancer	COSMIC
3	ARNTL	S27Y (0.74±0.14)	large_intestine, cancer	COSMIC
4	ARNTL	R37C (0.76±0.14)	large_intestine, cancer	COSMIC
5	ARNTL	R37H (0.76±0.14)	large_intestine, cancer	COSMIC
6	ARNTL	E62Q (0.778±0.079)	oesophagus, cancer	COSMIC
7	ARNTL	E65K (0.770±0.070)	genital_tract, cancer	COSMIC
8	ARNTL	H66P (0.760±0.065)	thyroid, cancer	COSMIC
9	ARNTL	I80F (0.76±0.11)	thyroid, cancer	COSMIC
10	ARNTL	R83Q (0.69±0.13)	liver, cancer	COSMIC
11	ARNTL	R84H (0.68±0.11)	stomach, cancer	COSMIC
12	ARNTL	R85Q (0.65±0.14)	ovary, cancer	COSMIC
13	ARNTL	M88I (0.58±0.15)	NS, cancer	COSMIC
14	ARNTL	S90I (0.50±0.12)	haematopoietic_and_lymphoid_tissue, cancer	COSMIC
15	ARNTL	A104T (0.29±0.10)	central_nervous_system, cancer	COSMIC
16	ARNTL	D110Y (0.301±0.072)	large_intestine, cancer	COSMIC
17	ARNTL	T140S (0.23±0.13)	breast, cancer	COSMIC
18	ARNTL	D145N (0.17±0.12)	large_intestine, cancer	COSMIC
19	ARNTL	D145E (0.17±0.12)	pancreas, cancer	COSMIC
20	ARNTL	V162I (0.033±0.022)	biliary_tract, cancer	COSMIC
21	ARNTL	R166G (0.045±0.032)	breast, cancer	COSMIC
22	ARNTL	Q190E (0.127±0.076)	upper_aerodigestive_tract, cancer	COSMIC
23	ARNTL	P198L (0.187±0.097)	skin, cancer	COSMIC
24	ARNTL	T224S (0.58±0.18)	stomach, cancer	COSMIC
25	ARNTL	P234H (0.54±0.17)	skin, cancer	COSMIC
26	ARNTL	R238Q (0.49±0.21)	large_intestine, cancer	COSMIC
27	ARNTL	R244Q (0.47±0.24)	large_intestine, cancer	COSMIC
28	ARNTL	S246C (0.48±0.24)	cervix, cancer	COSMIC
29	ARNTL	C249R (0.50±0.29)	thyroid, cancer	COSMIC
30	ARNTL	V260A (0.60±0.22)	large_intestine, cancer	COSMIC
31	ARNTL	P292T (0.41±0.16)	pancreas, cancer	COSMIC
32	ARNTL	D299Y (0.580±0.056)	liver, cancer	COSMIC
33	ARNTL	A345T (0.120±0.045)	liver, cancer	COSMIC
34	ARNTL	S372L (0.084±0.056)	breast, cancer	COSMIC
35	ARNTL	E375G (0.084±0.056)	large_intestine, cancer	COSMIC

2) **Graphical presentation of the NetPhos 3.1 server phosphorylation sites prediction for hAHR, hAHRR, hSIM1, hSIM2, hHIF2a, hNPAS4, hARNT2, and hBMAL1**.

hAHR

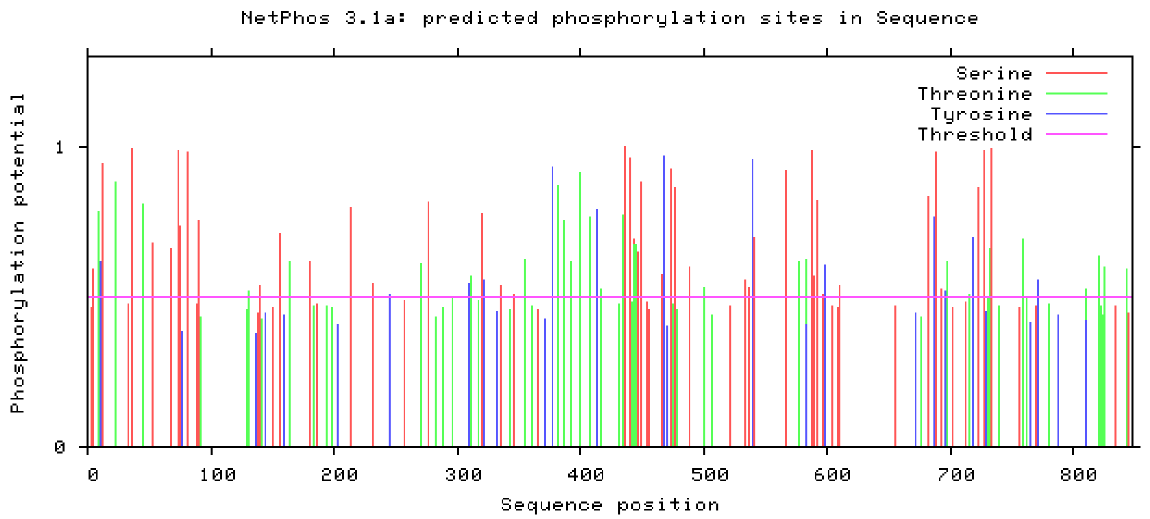

hAHRR

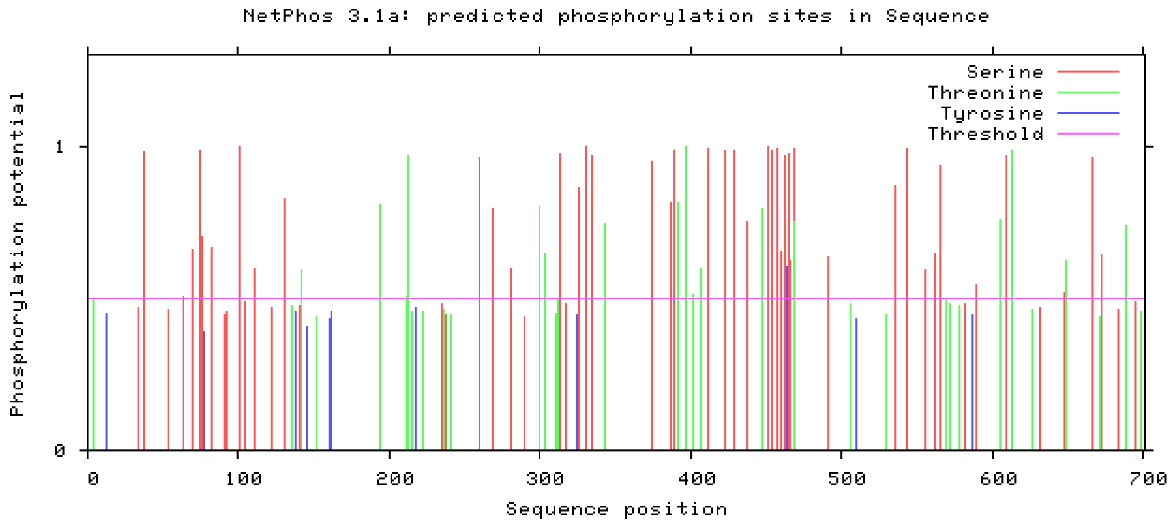

hSIM1

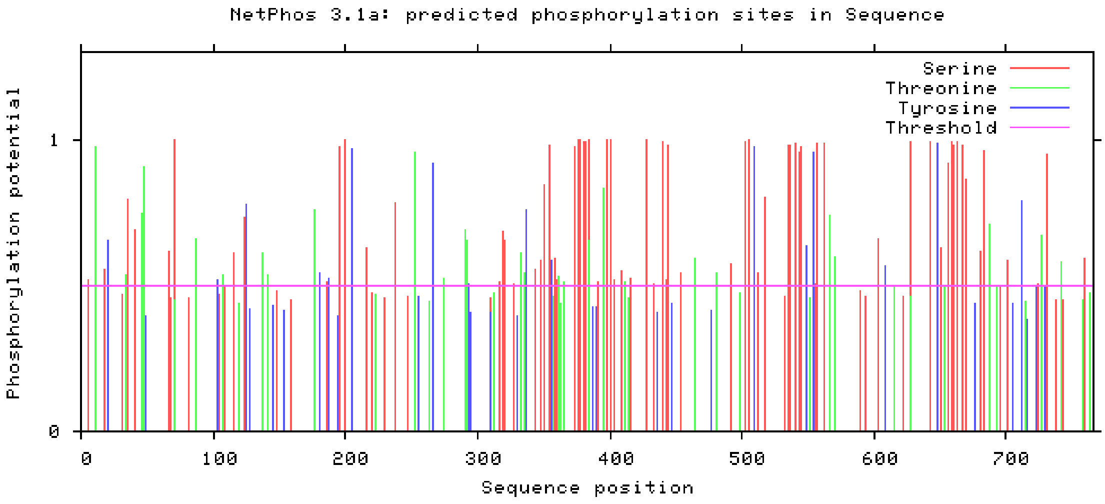

hSIM2

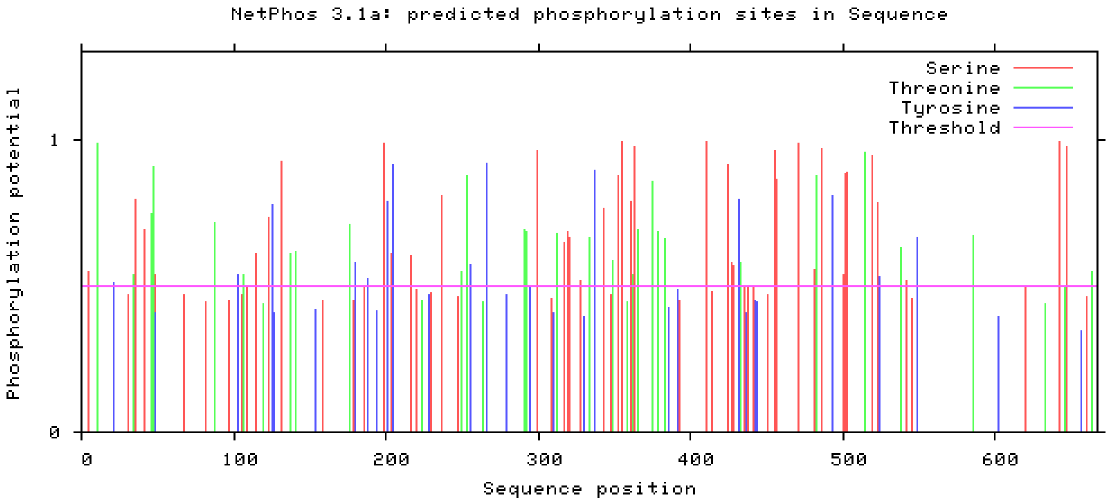

hHIF2a

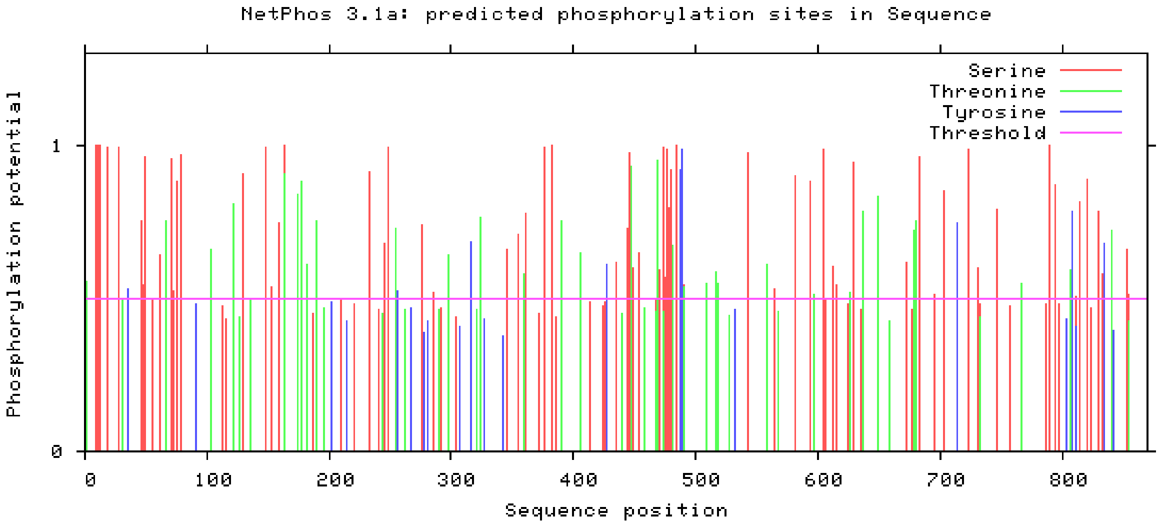

hNPAS4

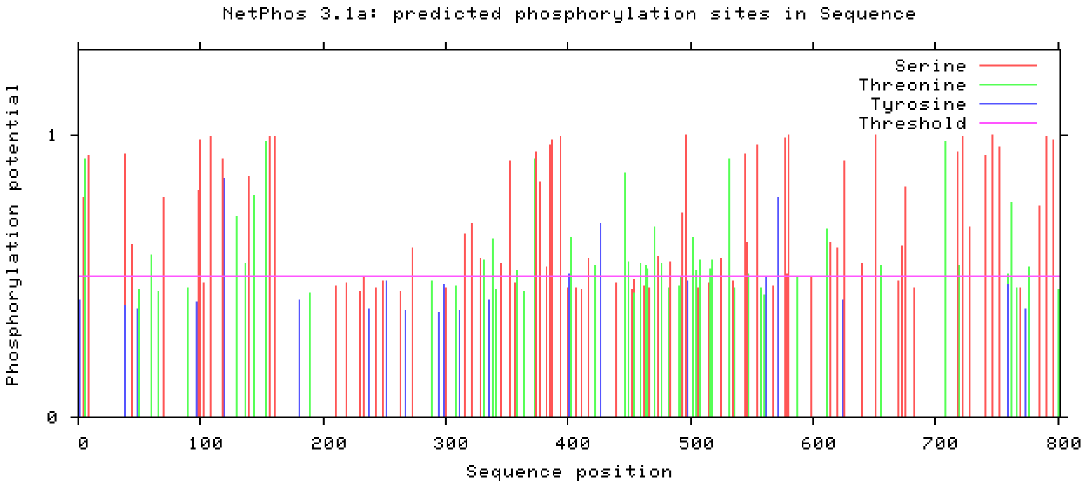

hARNT2

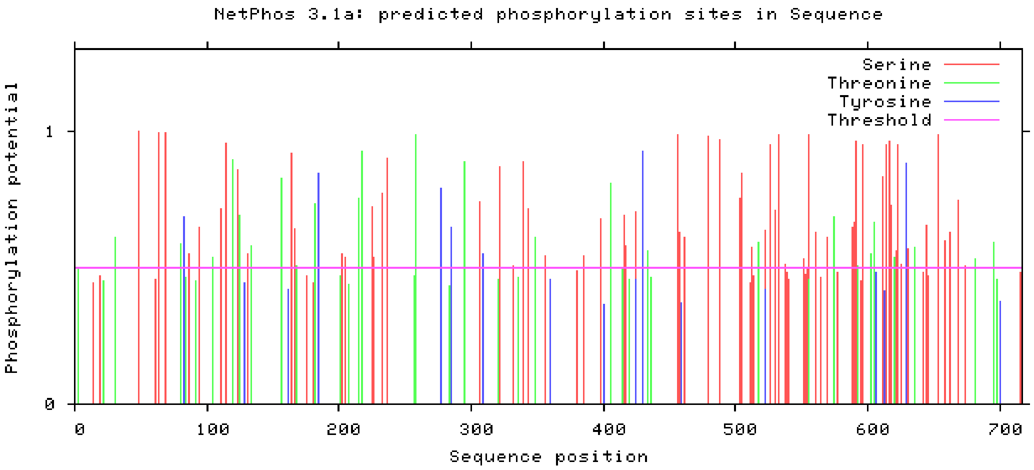

hBMAL1

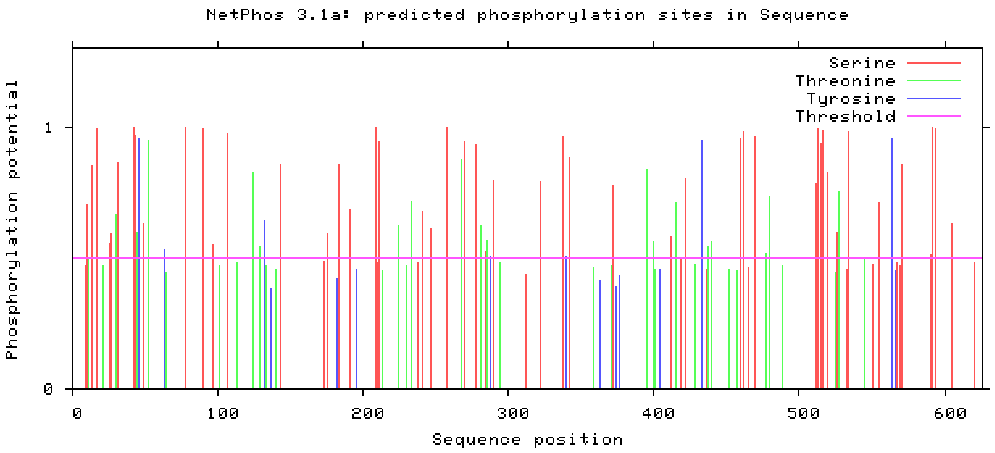

2) STRING-based external interactome of selected bHLH-PAS TFs with the "first shell" interactors. The confidence level of 0.7 was used in this analysis

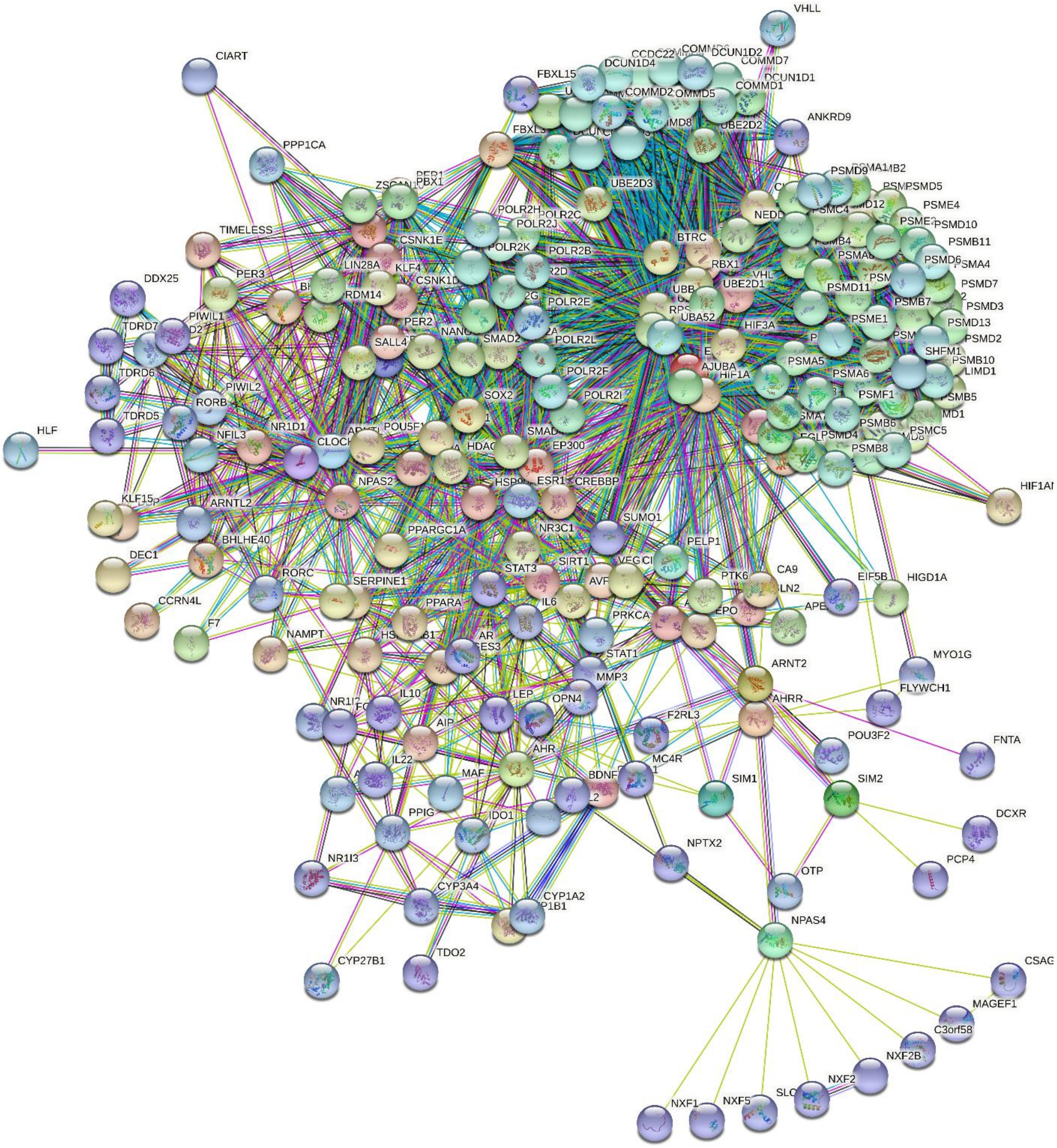


```
Confidence: 0.7
number of nodes:  223
number of edges:  3721
average node degree:    33.4
avg. local clustering coefficient:  0.736
expected number of edges:     1216
PPI enrichment p-value: < 1.0e-16
```

3) Plots of STRING analysis of individual proteins: hAHR, hAHRR, hSIM1, hSIM2, hHIF2a, hNPAS4, hARNT2, and hBMAL1

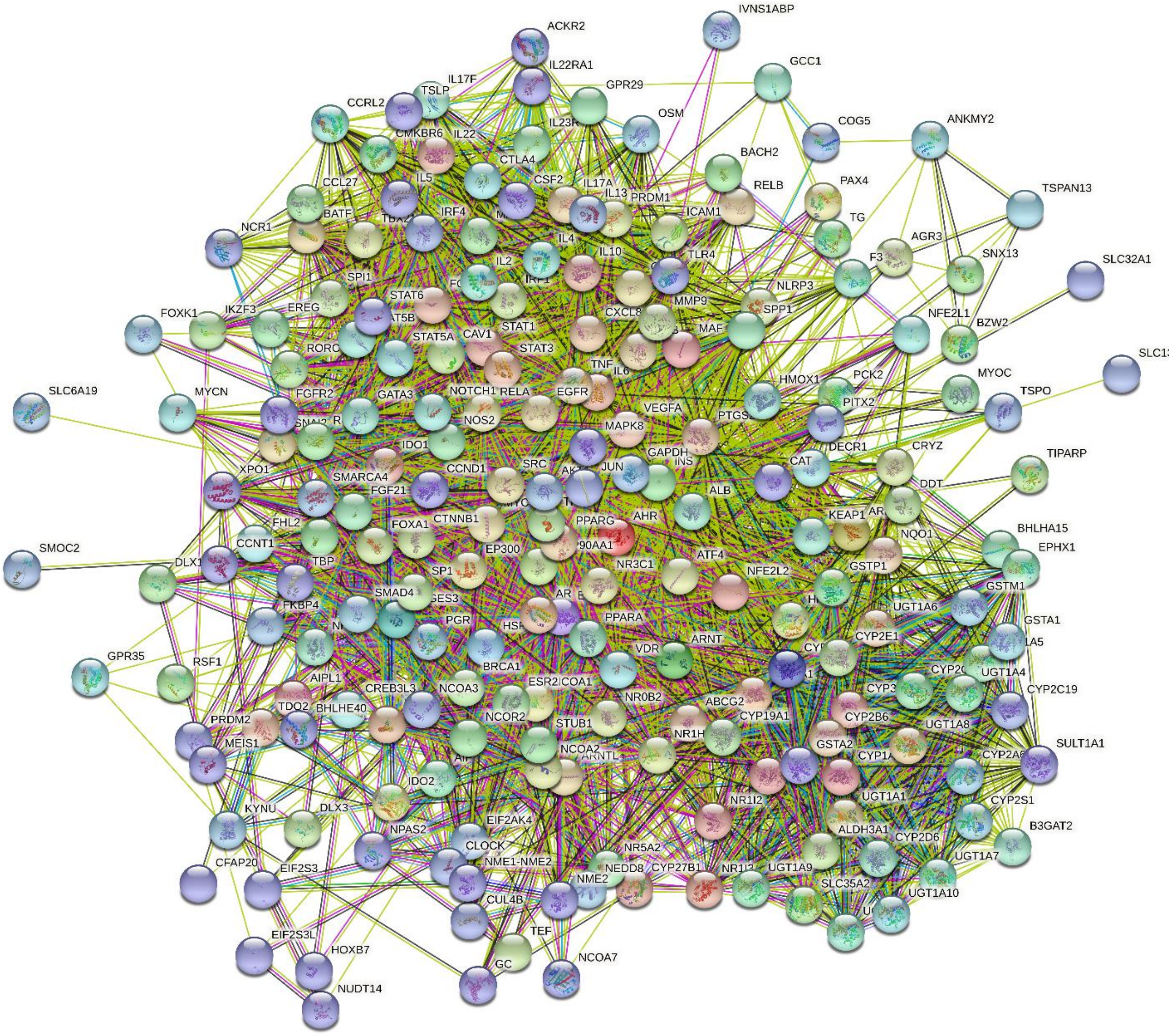

number of nodes:	207
number of edges:	4071
average node degree:	39.3
avg. local clustering coefficient:	0.66
expected number of edges:	1367
PPI enrichment p-value:	< 1.0e-16

STRING-based external interactome of AhR with "first shell" interactors with moderate confidence level of 0.4 and corresponding description of this PPI.

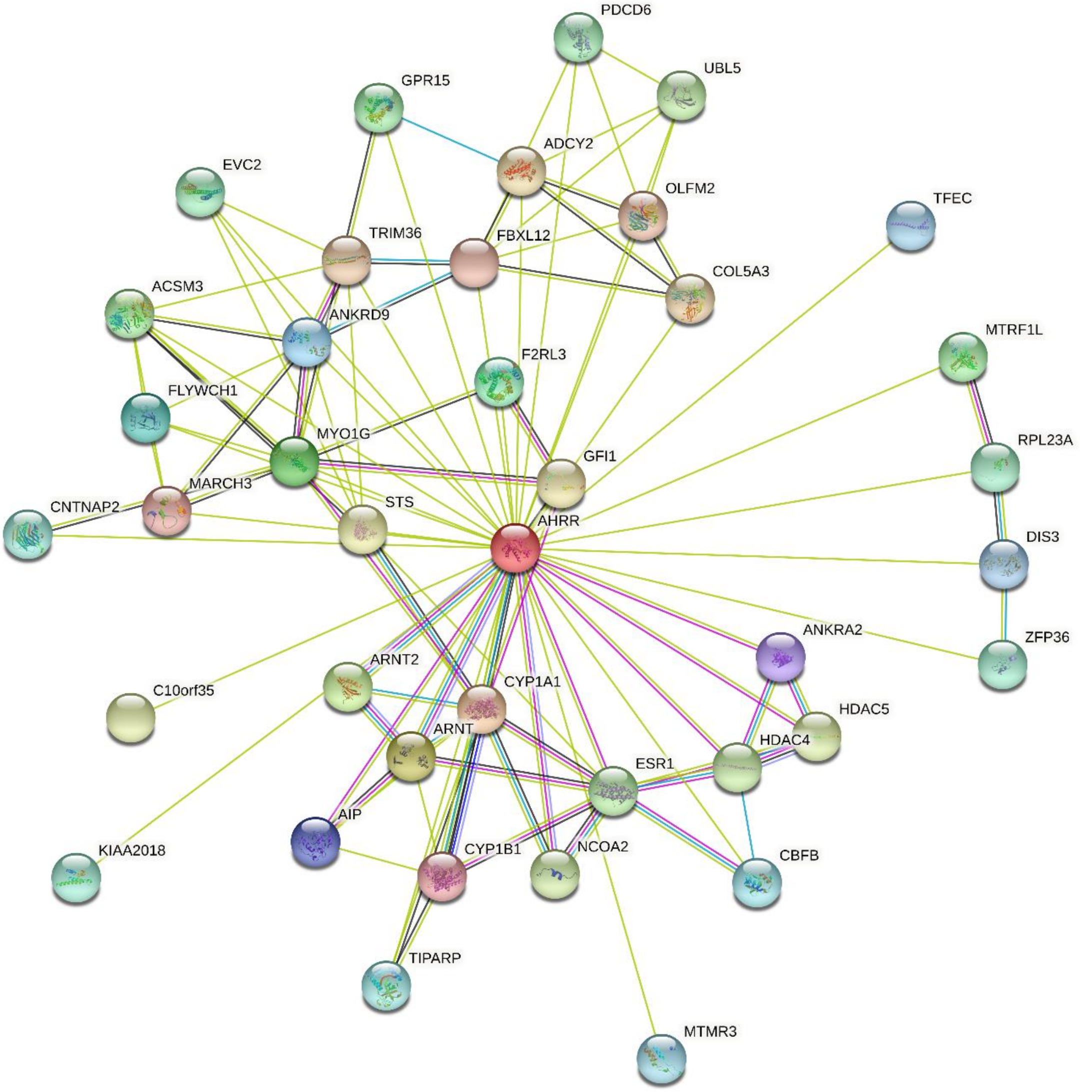

Confidence: 0.4
number of nodes: 39
number of edges: 105
average node degree: 5.38
avg. local clustering coefficient: 0.809
expected number of edges: 48
PPI enrichment p-value: 1.01e-12

STRING-based external interactome of AhRR with "first shell" interactors with moderate confidence level of 0.4 and corresponding description of this PPI.

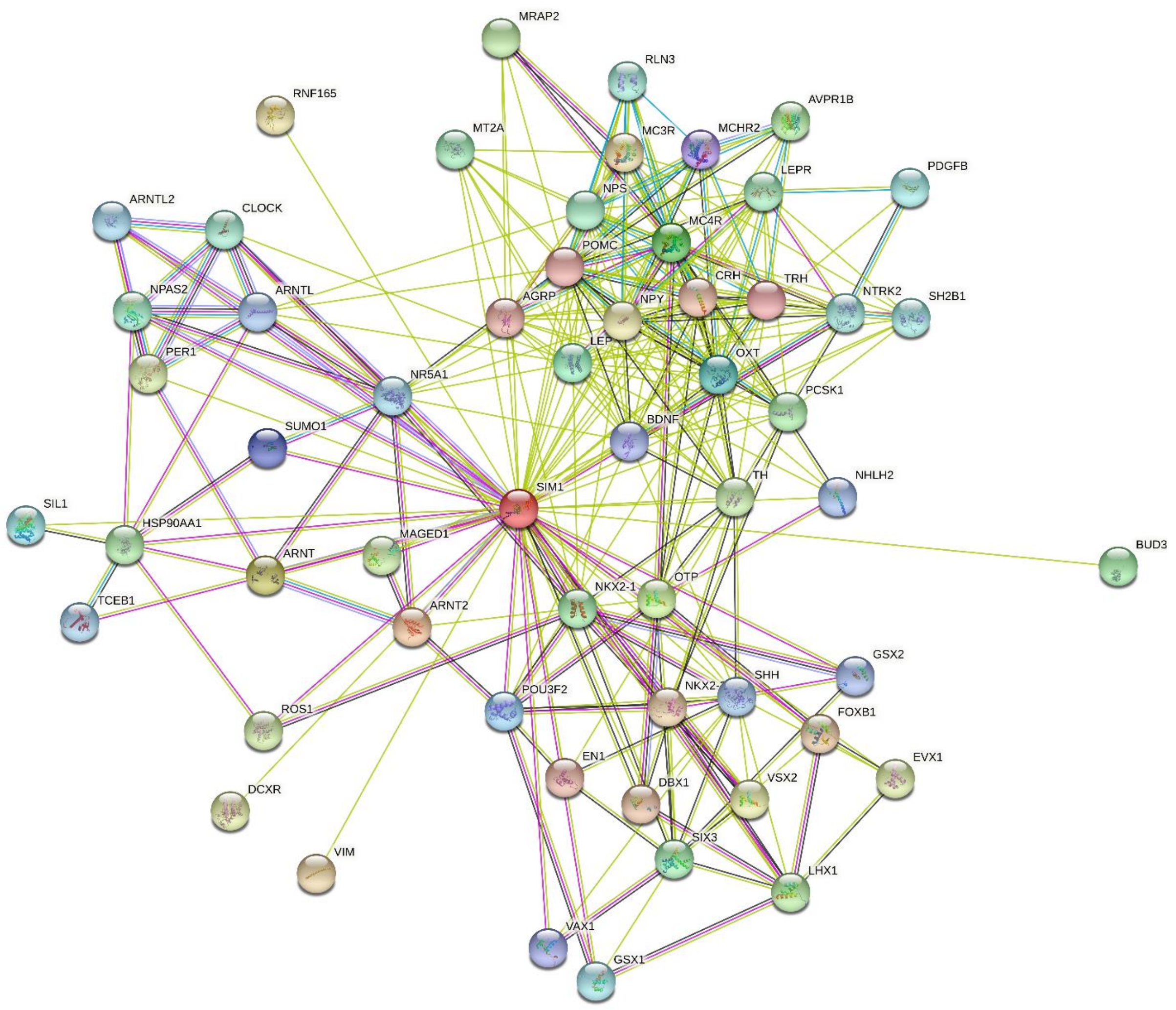

Confidence: 0.4	
Number of nodes:	57
number of edges:	288
average node degree:	10.1
avg. local clustering coefficient:	0.713
expected number of edges:	92
PPI enrichment p-value:	< 1.0e-16

STRING-based external interactome of SIM1 with "first shell" interactors with moderate confidence level of 0.4 and corresponding description of this PPI.

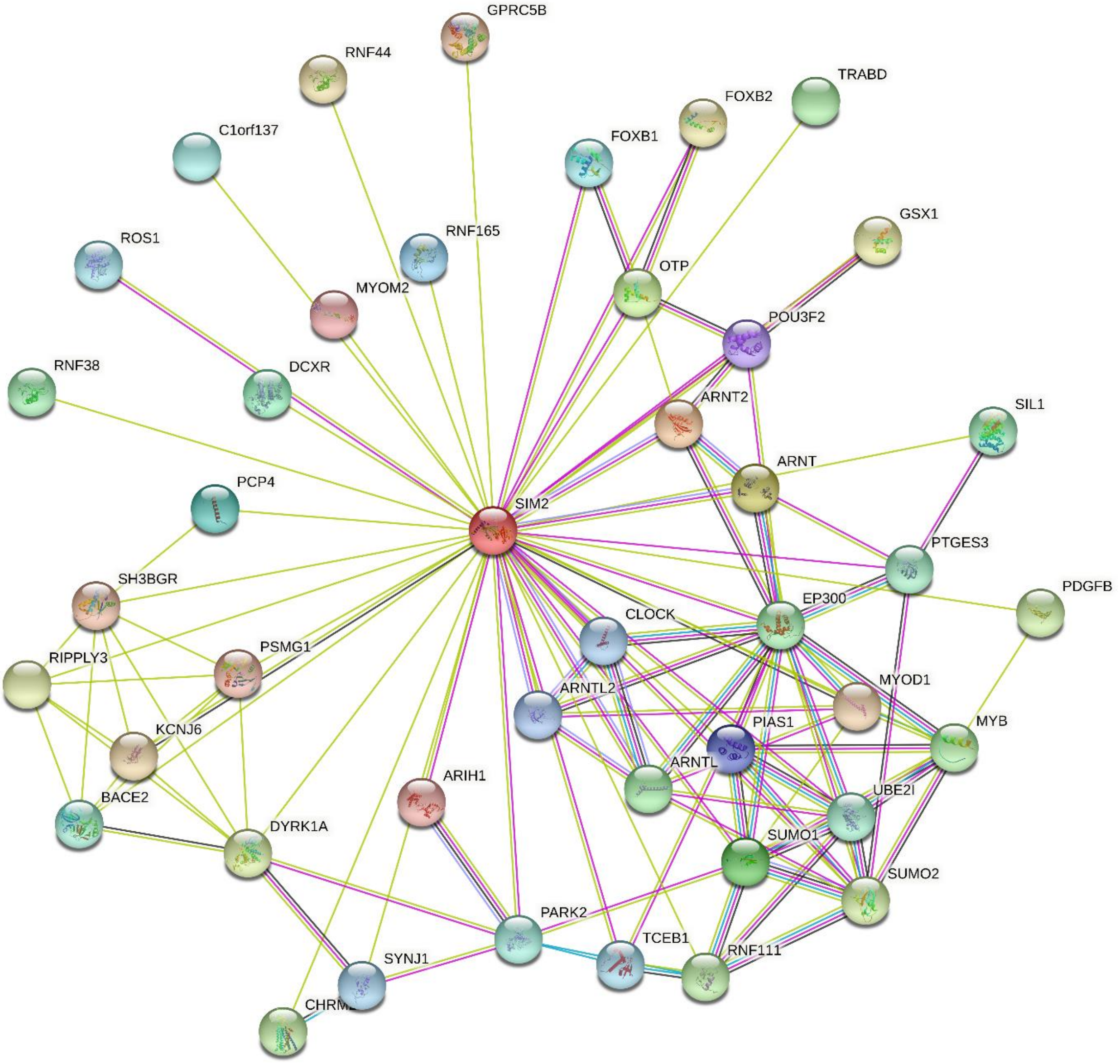

Confidence: 0.4

number of nodes:	43
number of edges:	114
average node degree:	5.3
avg. local clustering coefficient:	0.826
expected number of edges:	58
PPI enrichment p-value:	3.75e-11

STRING-based external interactome of SIM2 with "first shell" interactors with moderate confidence level of 0.4 and corresponding description of this PPI.

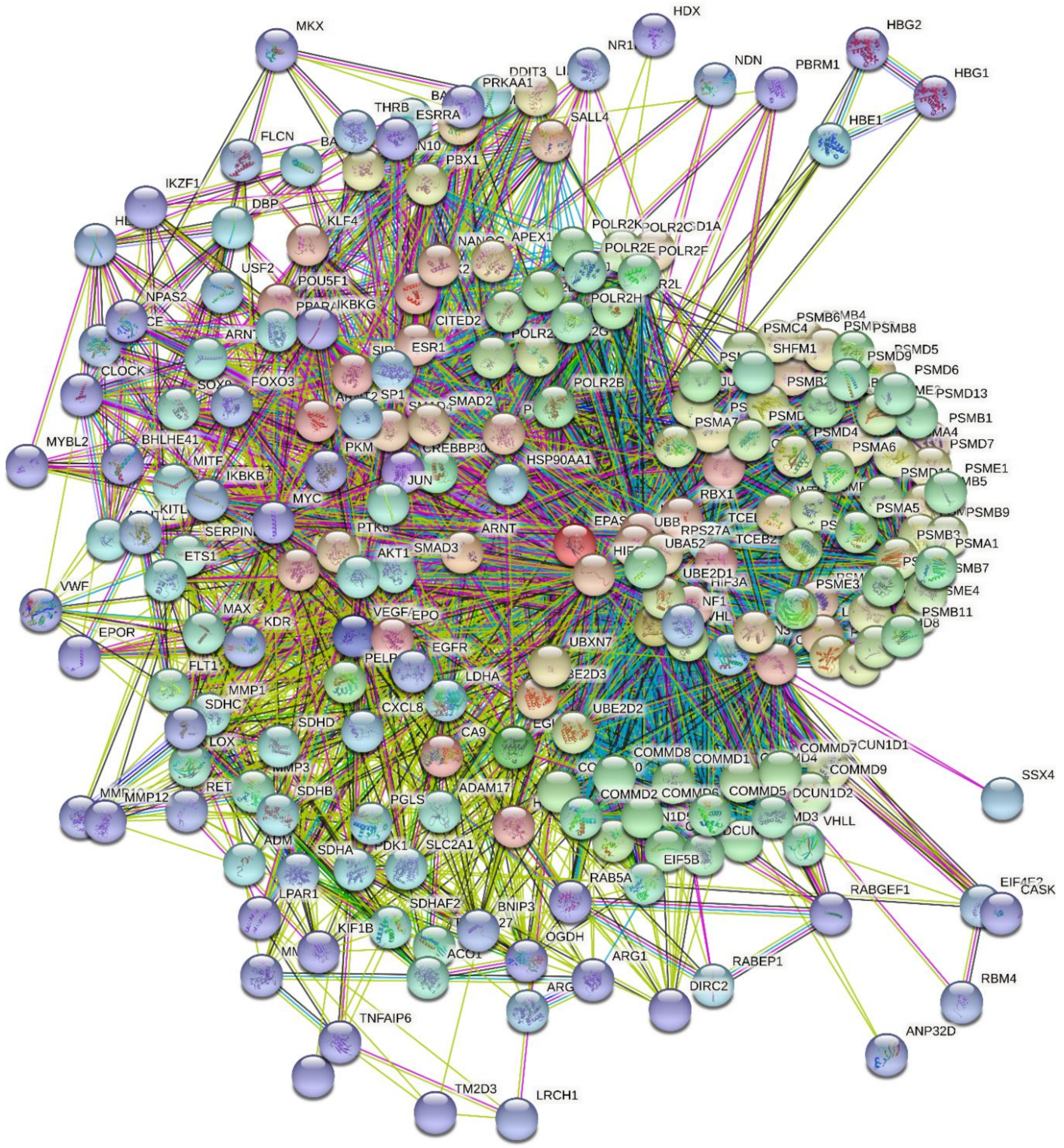

Confidence: 0.4
number of nodes: 213
number of edges: 4257
average node degree: 40
avg. local clustering coefficient: 0.74
expected number of edges: 1514
PPI enrichment p-value: < 1.0e-16

STRING-based external interactome of Hif-2α (EPAS1) with "first shell" interactors with moderate confidence level of 0.4 and corresponding description of this PPI.

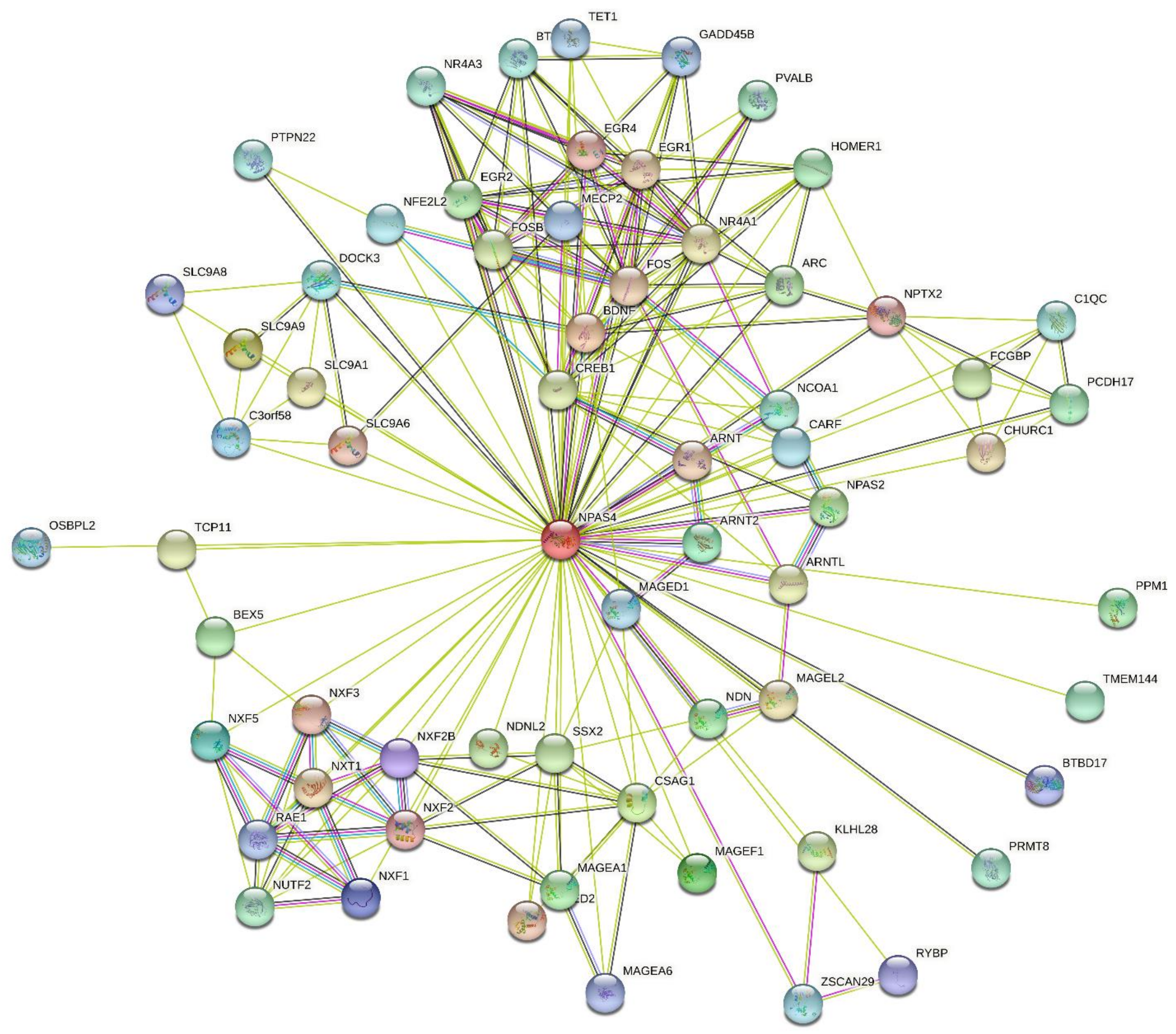

Confidence: 0.4
number of nodes: 64
number of edges: 223
average node degree: 6.97
avg. local clustering coefficient: 0.771
expected number of edges: 79
PPI enrichment p-value: < 1.0e-16

STRING-based external interactome of NPAS4 with "first shell" interactors with moderate confidence level of 0.4 and corresponding description of this PPI.

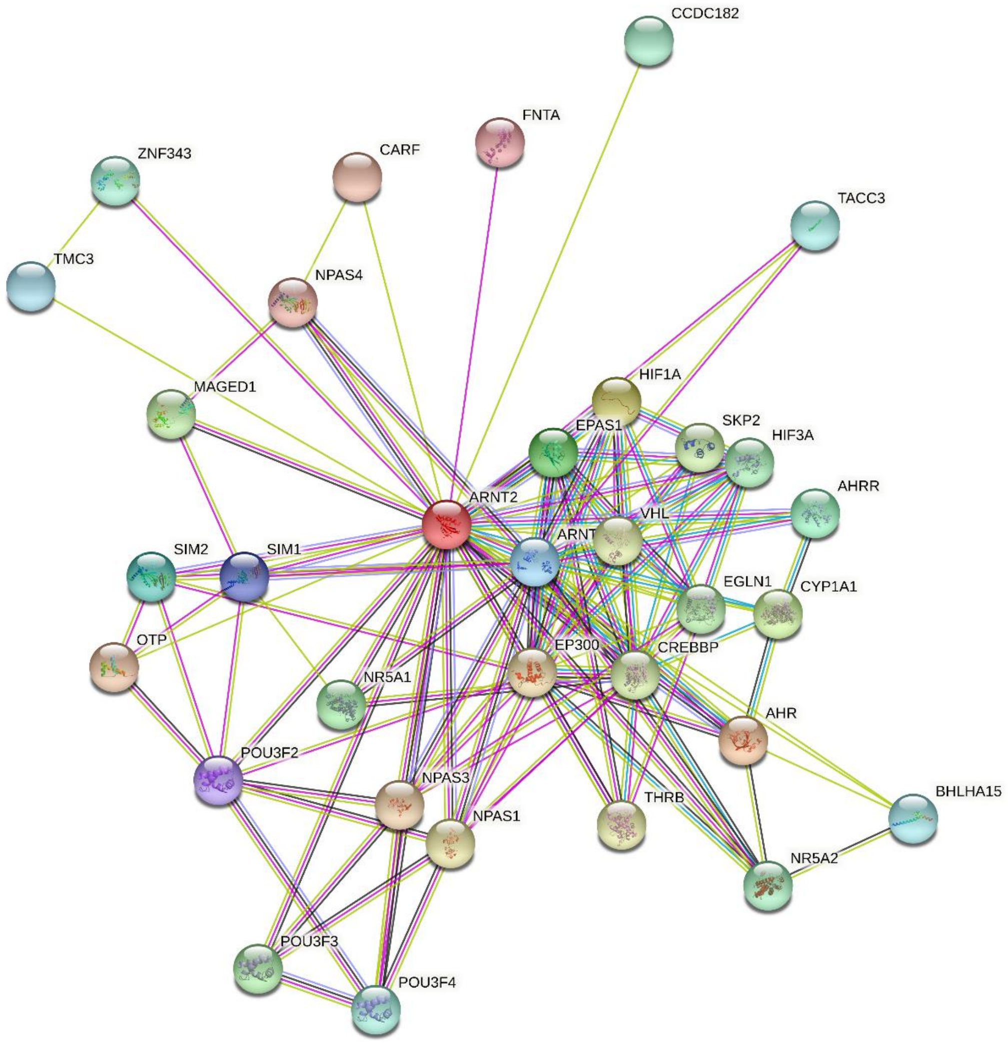

Confidence: 0.4
number of nodes: 33
number of edges: 117
average node degree: 7.09
avg. local clustering coefficient: 0.759
expected number of edges: 40
PPI enrichment p-value: < 1.0e-16

STRING-based external interactome of ARNT2 with "first shell" interactors with moderate confidence level of 0.4 and corresponding description of this PPI.

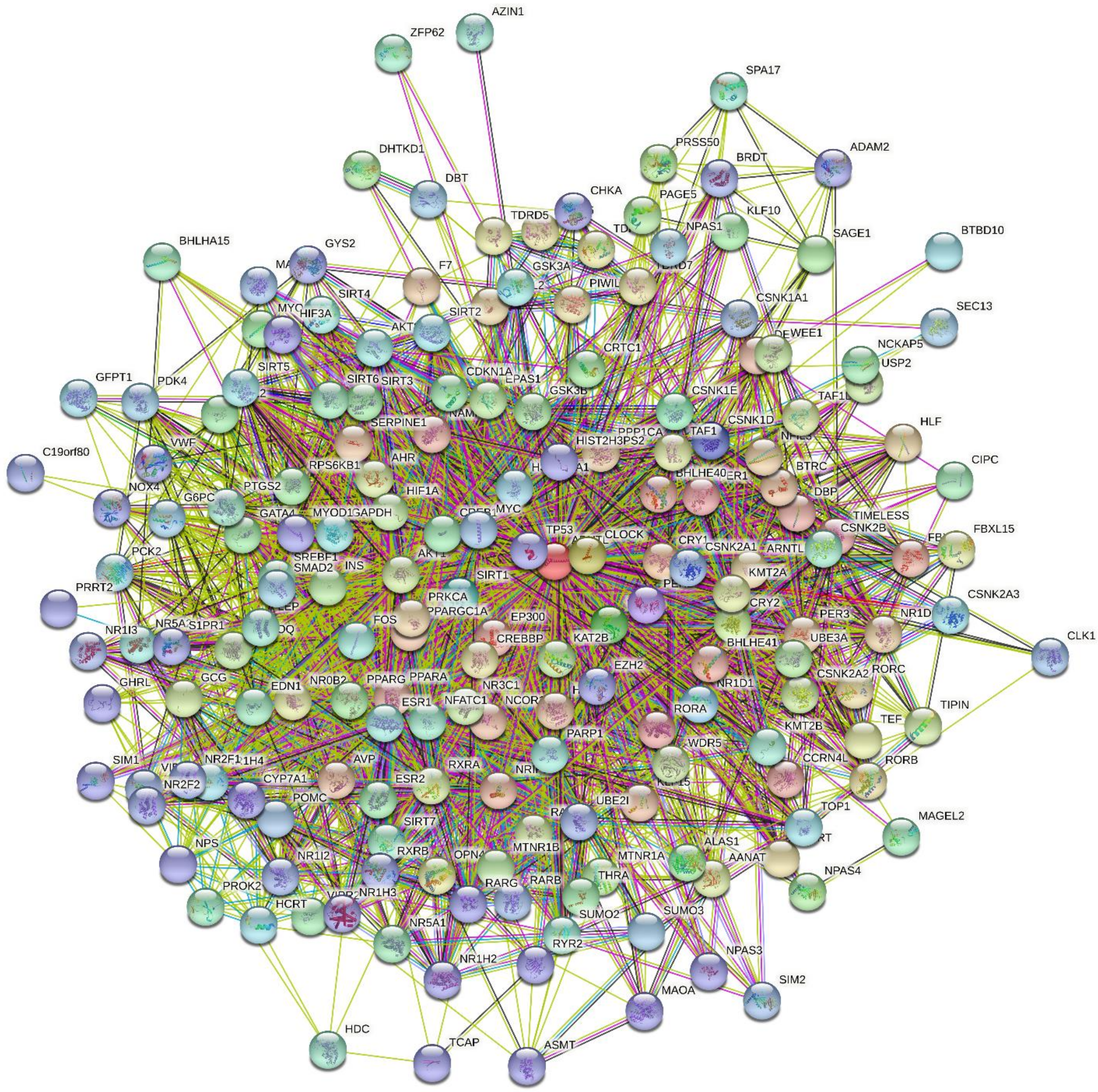

Confidence: 0.4
number of nodes: 181
number of edges: 2478
average node degree: 27.4
avg. local clustering coefficient: 0.618
expected number of edges: 782
PPI enrichment p-value: < 1.0e-16

STRING-based external interactome of BMAL1 (ARNTL) with "first shell" interactors with moderate confidence level of 0.4 and corresponding description of this PPI.
